# Intracellular Mechanical Stress‐Mediated Autophagy Cell Death via Nanospikes for Cancer Treatment

**DOI:** 10.1002/advs.202512256

**Published:** 2025-10-13

**Authors:** Yingze Li, Zihan Guo, Jiawei Fan, Ruimei Zhou, Jiayan Li, Zhixiang Hu, Weicheng Gu, Mengge Zheng, Chang Xu, Yichao Tang, Chang Chen, Yu Cheng

**Affiliations:** ^1^ Department of Thoracic Surgery Shanghai Pulmonary Hospital Tongji University School of Medicine Shanghai 200433 China; ^2^ Translational Research Institute of Brain and Brain‐Like Intelligence Shanghai Fourth People's Hospital School of Medicine Tongji University Shanghai 200434 China; ^3^ State Key Laboratory of Autonomous Intelligent Unmanned Systems Tongji University Shanghai 201210 China; ^4^ Collaborative Innovation Center for Brain Science Tongji University Shanghai 200092 China; ^5^ School of Mechanical Engineering Tongji University Shanghai 200092 China; ^6^ Institute of Acoustics School of Physics Science and Engineering Tongji University Shanghai 200092 China; ^7^ Central Laboratory Shanghai Pulmonary Hospital Tongji University School of Medicine Shanghai 200433 China

**Keywords:** autophagic cell death, lysosomal damage, mechanical drugs, mechanical stress, nanospikes

## Abstract

Mechanical signals are fundamental regulators of cell fate, yet how cells respond to mechanical stress at the subcellular level remains unclear. Inspired by natural spiky structures that concentrate mechanical stress at the nanoscale, a series of tunable gold nanospikes are designed to promote internalization and modulate mechanical stress intracellularly. The nanospikes with a length of 254.2 nm induced the highest cancer cell death compared to those with 104.0 and 45.4 nm. Mechanistically, nanospikes are internalized into lysosomes and triggered extensive lysosomal membrane disruption. Finite element simulations reveal that the tip stress generated by nanospikes with a length of 254.2 nm achieves the highest value within 5.233 to 9.902 kPa range across the majority of lysosome sizes, exceeding the mechanical threshold for lysosomal rupture. This mechanical stress on lysosomal membranes triggered autophagic cell death through the Galectin‐3 (Gal3)‐Trim16 signaling axis, establishing a direct mechanobiological link between nanostructure geometry and cell fate. Importantly, the nanospikes achieve 77.8% tumor inhibition, while the in situ melting via nanosecond pulsed laser enables reduced mechanical stress and attenuated cytotoxicity. This bioinspired morphological strategy provides a controllable method for tuning intracellular mechanics, providing new insights for the rational design of mechanical drugs for cancer treatment.

## Graphical abstract

Nanospikes with tunable morphology at the nanoscale induce lysosomal membrane damage by generating localized mechanical stress, activating Galectin‐3 (Gal3)‐Trim16‐mediated autophagic cell death. Finite element analysis and laser‐triggered spike ablation enable precise control of intracellular force signaling and cytotoxicity, offering a mechanoresponsive strategy for cancer treatment.

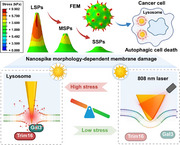

## 1 Introduction

Mechanical signals are increasingly recognized as critical regulators of biochemical signaling in various cellular functions.^[^ ^1^, ^2^, ^3^ ^]^ Over the past decades, numerous studies have elucidated how cells sense and transduce mechanical cues at the cellular and tissue levels, revealing their fundamental roles in the progression of major diseases such as cancer.^[^ ^4^, ^5^, ^6^ ^]^ However, increasing evidence suggests that intracellular mechanical signals represent a more central player of regulation, capable of fine‐tuning cellular behavior at the subcellular level.^[^ ^7^, ^8^, ^9^ ^]^ Nevertheless, how mechanical forces affect organelles, particularly how these forces are perceived and transduced into intracellular biochemical responses, remains unclear. Therefore, there is an urgent need for precision tools capable of modulating intracellular mechanical forces to better understand and apply subcellular mechanobiology in therapeutic contexts.

In nature, spiky structures are evolutionarily optimized to concentrate mechanical loading at their sharp tips, thereby exerting focused forces on contacted surfaces and inducing localized deformation.^[^ ^10^, ^11^ ^]^ These forces generate significant mechanical stress within the contacted membranes and, in turn, regulate membrane integrity and membrane‐dependent cellular activities.^[^ ^12^, ^13^ ^]^ Inspired by such biomechanical strategies, artificial spiky microparticles and nanoparticles have been developed in the biomedical field, particularly for cancer therapy.^[^ ^14^, ^15^ ^]^ For example, engineered structures such as nanoneedles, carbon nanotubes, and biocompatible devices could physically penetrate cell membranes to induce cancer cell death.^[^ ^16^, ^17^, ^18^ ^]^ This strategy provides a unique opportunity to induce force‐driven biological effects independent of traditional biochemical therapeutics, offering new avenues for mechanical anticancer regulation.

Despite these promising advances, the mechanistic understanding of how spiky nanostructures influence intracellular organelles remains incomplete. Lysosomes, which serve as central regulators of intracellular biochemical signaling, are highly sensitive to mechanical signals, and maintaining their homeostasis is essential for cell viability.^[^ ^19^, ^20^, ^21^ ^]^ The reported spiky nanoparticles often act as drug carriers for enhanced cellular uptake and create defects in the lysosomal membrane to increase transfection efficiency.^[^ ^22^, ^23^ ^]^ By themselves, they did not cause a significant anticancer effect in vivo, which needs to be combined with other therapeutic approaches.^[^ ^24^, ^25^, ^26^ ^]^ It should be noted that the precise mechanical stress membrane interactions that trigger lysosomal membrane permeabilization (LMP) may play as a key initiating event of programmed cell death, which have not yet been quantitatively defined. In addition, the molecular pathways that link mechanically induced lysosomal damage to cell fate decisions remain unexplored. Further strategies are needed to enhance the in vivo anticancer efficacy of nanospikes.

In this study, we developed bioinspired spiky gold nanoparticles by growing tunable gold nanospikes on the iron oxide nanoparticle (IO)‐based cores, achieving precise control over spike length and density via silver ion‐mediated synthesis.^[^ ^27^ ^]^ We demonstrated that nanospikes with tunable length were preferentially internalized into the lysosomes of cancer cells, where they exerted localized mechanical forces that generated significant membrane stress, leading to extensive lysosomal disruption (**Scheme** 1). Finite element simulations suggested that such stress could exceed the threshold required for LMP, thereby offering a mechanistic basis for downstream cellular effects. Mechanistically, we explored the potential of nanospikes with the tunable length to induce LMP for cancer cell death, revealing a force‐sensitive regulatory pathway downstream of lysosomal stress. In vivo, these nanospikes could effectively suppress tumor growth, while the incorporation of nanosecond pulsed laser irradiation enabled selective ablation of intracellular gold spikes, allowing real‐time control of cytotoxicity within the tumor microenvironment. Furthermore, immunofluorescence and immunohistochemistry staining further confirmed the molecular mechanism of LSP‐mediated autophagy‐dependent cell death in tumor tissues in vivo. This strategy represents a controllable synthetic approach for mechanical drugs, offering a promising avenue for integrating intracellular mechanical stress‐based interventions into future cancer treatment paradigms.

## 2 Results

### 2.1 Cytotoxicity of Gold Nanospikes Is Critically Dependent on the Spike Length

**Figure 1:**
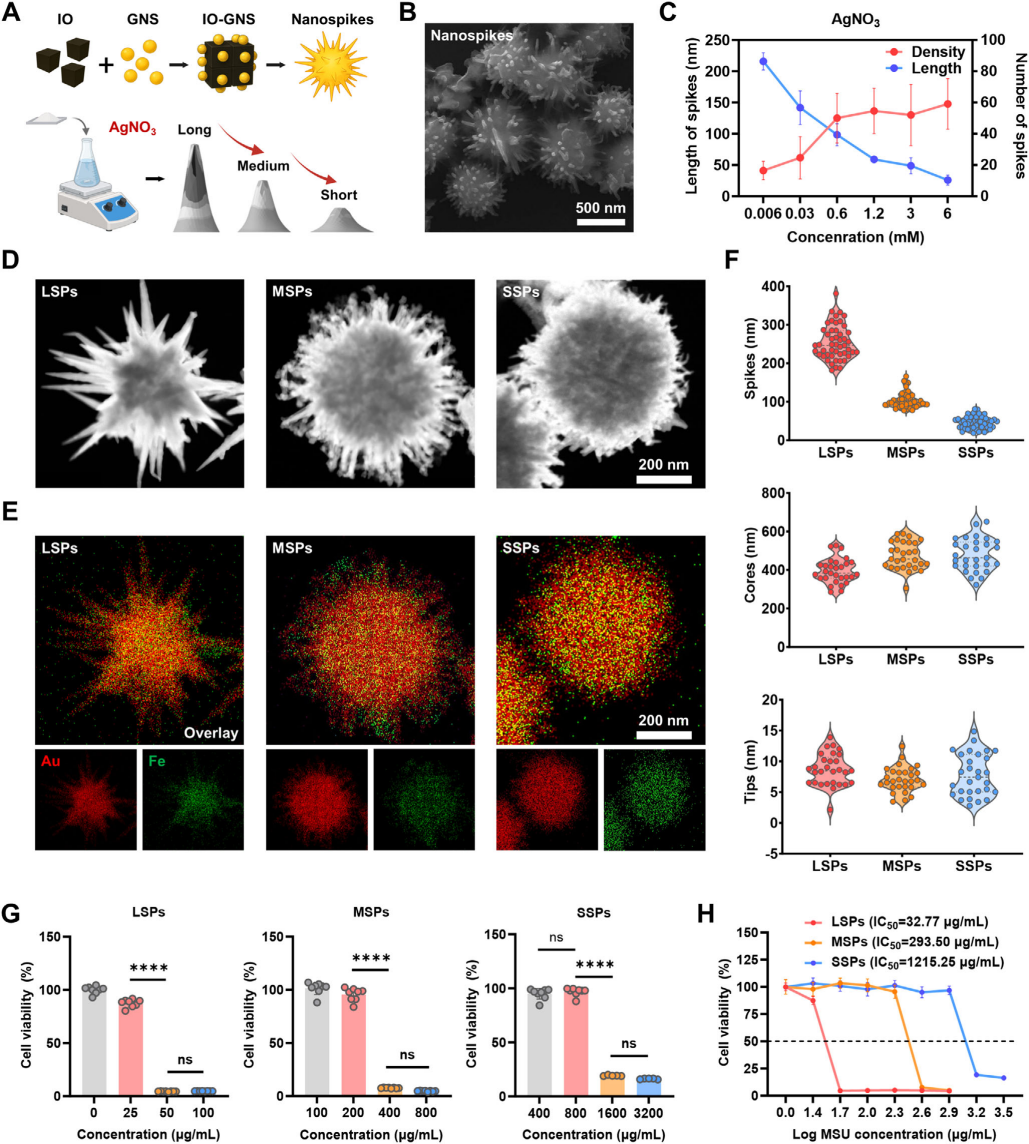
Nanospikes with different spike lengths exhibit distinct physicochemical characteristics and cytotoxicity profiles. A) Schematic illustration of the synthesis of LSPs, MSPs, and SSPs by controlling AgNO_3_ concentrations. B–D) SEM images of gold nanoparticles (B), with quantification of spike length and number as a function of AgNO_3_ concentration (C), and transmission electron microscopy (TEM) images morphology of LSPs, MSPs, and SSPs (D). E) Elemental mapping images of gold (Au) and iron (Fe) distribution in LSPs, MSPs, and SSPs. F) The size distribution of spikes, core diameters, and tip lengths in the three particle types. G–H) Cell viability of LLC cells after 24 h incubation with LSPs, MSPs, and SSPs at various Au concentrations (G) and dose‐response curves with calculated IC_50_ values (H). Data are presented as mean ± s.d. Statistical significance: *p* < 0.05 (^*^), *p* < 0.01 (^**^), *p* < 0.001 (^***^), *p* < 0.0001 (^****^).

Drawing inspiration from natural spiky architectures, gold nanoparticles with finely tuned spike morphologies were successfully synthesized (**Figure** **1**A; Figure S1A,B, Supporting Information). Specifically, gold nanoseeds (GNS) were deposited onto IO cores to establish nucleation sites for spike formation. We identified that a GNS‐to‐IO core ratio of 1:2 yielded optimal spike density, spike distribution, as well as colloidal stability. Consequently, this ratio was adopted for all subsequent experiments (Figure S1C–E, Supporting Information). The spike formation on particle surfaces was confirmed via scanning electron microscopy (SEM) (Figure 1B).

To precisely regulate the morphology of nanospikes, the key reagents were systematically varied, including the gold precursor (HAuCl_4_), reducing agent (ascorbic acid, AA), and shape‐directing agents (silver nitrate, AgNO_3_; hydrochloric acid, HCl). A specific molar concentration of HAuCl_4_ (0.5 mm) and AA (1 mm) was found to be essential for maintaining stable spiky morphologies, as deviations from this concentration did not significantly alter spike length or density (Figure S1F,G, Supporting Information). Furthermore, the acidity of the reaction environment was modulated by HCl, which was crucial for spiky morphologies. A molar concentration of 20 mm HCl yielded optimal structures, with lower pH progressively enhancing spike length while moderately increasing density (Figure S1H, Supporting Information). Significantly, AgNO_3_ was identified as the most influential agent affecting spike density and length. Higher concentrations reduced spike length, but increased density, demonstrating an inverse correlation with length and a positive correlation with density (Figure 1C; Figure S1I, Supporting Information). Leveraging these insights, three distinct variants of gold nanospike were synthesized with long spiky nanoparticles (LSPs), medium spiky nanoparticles (MSPs), and short spiky nanoparticles (SSPs), each characterized by well‐distributed spikes and consistent core–shell compositions (Figure 1D).

Detailed elemental and structural analyses confirmed that gold comprised over 90% of the nanospike composition, with uniform distribution across the IO cores (Figure 1E; Figure S2A, Supporting Information). Optical characterization revealed distinct absorption peaks in the near‐infrared (NIR) region, suggesting that the length of nanospikes determined their physical properties (Figure S2B, Supporting Information). Surface charge measurements showed similar zeta potentials for LSPs, MSPs, and SSPs, ≈−13.5, −14.9, and −13.9 mV, respectively (Figure S2C, Supporting Information). Hydrated diameters remained consistent across the three kinds of nanospikes (Figure S2D, Supporting Information). Additionally, spike lengths were precisely quantified as 254.2 ± 44.3 nm (LSPs), 104.0 ± 20.2 nm (MSPs), and 45.4 ± 15.6 nm (SSPs), alongside comparable core sizes and tip diameters measuring below 10 nm across all variants (Figure 1F).

To further evaluate the cytotoxicity of nanospikes with different spike lengths, cell viability assays were performed on LLC cells. Significant differences in cytotoxic effects were observed among the three nanospikes. Surprisingly, all three types exhibited a sharp increase in cytotoxicity within specific concentration ranges (Figure 1G; Figure S2E, Supporting Information). For instance, LSPs demonstrated the strongest cytotoxic effect compared to MSPs and SSPs, with a pronounced decrease in LLC cell viability observed in the 25–50 µg mL^−1^ (based on Au concentration), resulting in approximately a 19.4‐fold reduction. Moreover, the calculated IC_50_ values were 32.77 µg mL^−1^ for LSPs, 293.50 µg mL^−1^ for MSPs, and 1215.26 µg mL^−1^ for SSPs, indicating a clear length‐dependent cytotoxic trend (Figure 1H). LDH release assays further confirmed LSPs’ superior potency, showing maximal cytotoxicity after coculture (Figure S2F, Supporting Information). These findings demonstrated the critical role of spike length in dictating anticancer efficacy, establishing engineered nanospikes as promising therapeutic agents for subsequent in vivo evaluation and mechanistic exploration.

### 2.2 Morphology‐Dependent Nanospikes Trigger Mechanical Disruption of the Lysosomal Membrane

**Figure 2:**
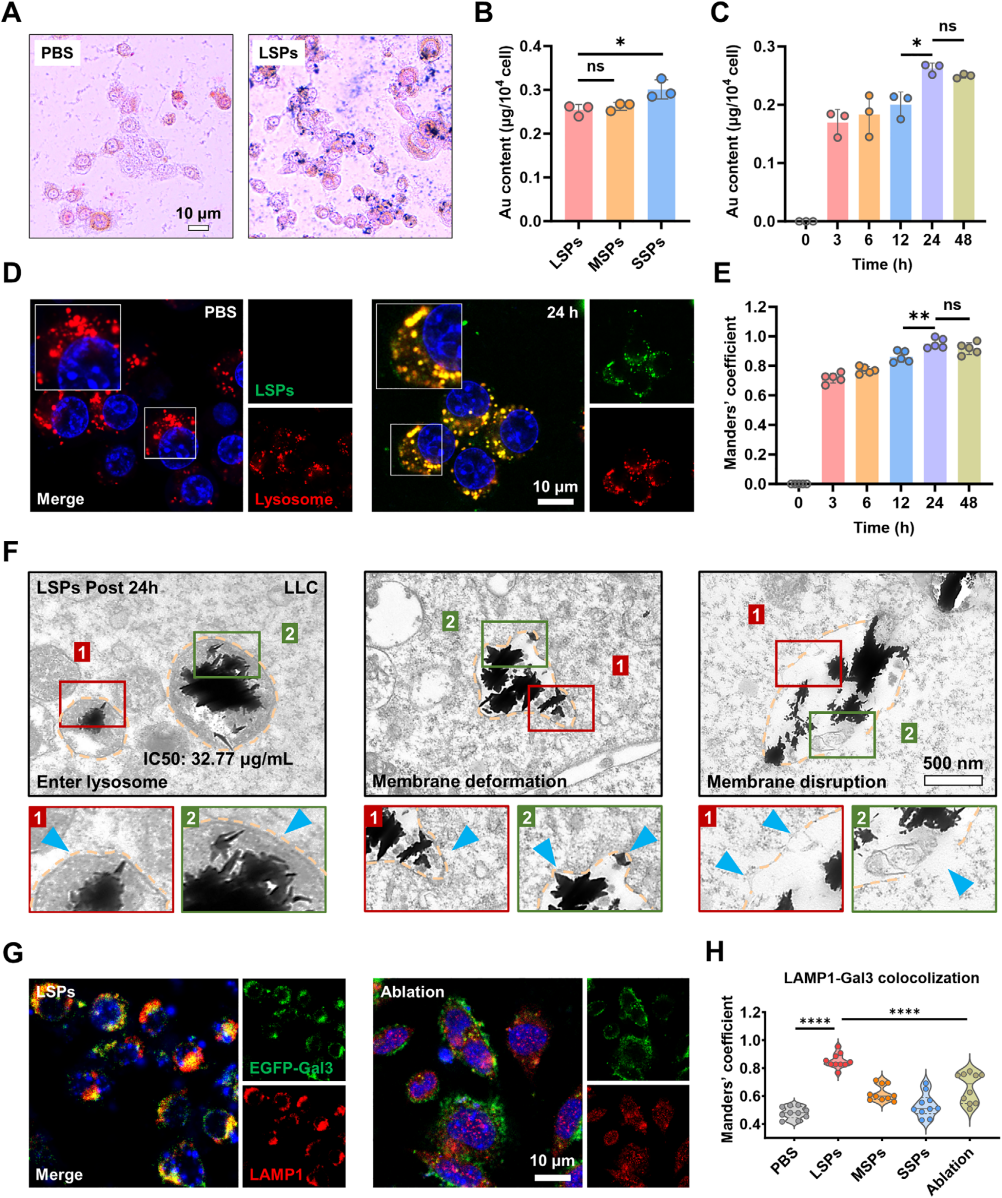
Programmable spike length of gold nanoparticles governs the degree of lysosomal damage in cancer cells. A) PB staining of LLC cells incubated with or without LSPs (32.77 µg mL^−1^). B,C) Au content in cells after treatment with LSPs, MSPs, or SSPs for 24 h (B), and time‐dependent Au uptake of LSPs (C) measured by ICP‐MS. Data are presented as mean ± s.d. Statistical significance: *p* < 0.05 (^*^), *p* < 0.01 (^**^), *p* < 0.001 (^***^), *p* < 0.0001 (^****^). D,E) Confocal fluorescence imaging of LSPs (green) colocalized with lysosomes (red) in LLC cells, with nuclei stained by Hoechst (blue) (D), and quantification of colocalization at different time points using Manders’ coefficient (E). Data are presented as mean ± s.d. Statistical significance: *p* < 0.05 (^*^), *p* < 0.01 (^**^), *p* < 0.001 (^***^), *p* < 0.0001 (^****^). F) Subcellular distribution of LSPs (32.77 µg mL^−1^) in lysosomes of LLC cells after co‐culture for 24 h by bio‐TEM. G,H) Confocal fluorescence imaging of Gal3 (green) colocalized with lysosomes (red) (G) and quantification by Manders’ coefficient (H) in EGFP‐Gal3‐transfected cells treated with PBS, LSPs, MSPs, SSPs, and LSP plus laser ablation. Lysosomes were labeled with LAMP1 antibody (red). Data are presented as mean ± s.d. Statistical significance: *p* < 0.05 (^*^), *p* < 0.01 (^**^), *p* < 0.001 (^***^), *p* < 0.0001 (^****^).

Given the significant differences in the cancer cell‐killing efficiencies of LSPs, MSPs, and SSPs, we proceeded to investigate their interactions with LLC cells. Prussian blue (PB) staining and inductively coupled plasma (ICP) analysis revealed substantial uptake of all three nanospikes by LLC cells (**Figure** **2**A,B). Notably, SSPs exhibited the highest internalization efficiency compared with LSPs and MSPs, yet displayed the highest IC50 value (1215.25 µg mL^−1^), indicating that spike length rather than uptake efficiency was the dominant factor driving cell death. A time‐dependent internalization was confirmed, with nanoparticle accumulation peaking at approximately 24 h post‐incubation, as quantified by ICP measurements (Figure 2C). Moreover, ≈80% of FITC‐labeled nanospikes (green) were located within lysosomes (red) after 24 h incubation, as confirmed by both LysoTracker Red staining and LAMP1 immunofluorescence, indicating the efficient lysosomal targeting effect (Figure 2D,E; Figure S3A‐C, Supporting Information). EDX analysis of bio‐TEM further confirmed that the nanospikes localized within lysosomes contained both Au and Fe elements, validating their internalization and lysosomal localization (Figure S3D, Supporting Information). To assess the impact of internalized nanospikes on lysosomal integrity, lysosomal changes were monitored at different time points using bio‐TEM. At 4 h, nanospikes were observed in contact with the plasma membrane or being internalized into lysosomes (Figure S3E, Supporting Information). By 24 h, significant lysosomal membrane deformation and rupture were evident, with nanospikes clustering inside and disrupting membrane integrity. The formation of autolysosomes and the discontinuities in the membrane structure were clearly observed, suggesting activation of autophagy in response to lysosomal damage (Figure 2F).

To determine whether lysosomal disruption and cytotoxicity were mechanically mediated by the spikes, we employed a nanosecond‐pulsed laser at 808 nm for melting LSPs (Figure S4A, Supporting Information). UV–vis analysis confirmed that a 2‐min exposure to nanosecond‐pulsed laser effectively ablated the gold spikes of LSPs, as evidenced by altered absorbance profiles (Figure S4B,C, Supporting Information). Besides, TEM imaging post‐ablation revealed significantly shortened and blunted spikes relative to untreated LSPs (Figure S4D–F, Supporting Information). Subsequent cell viability assays indicated that cytotoxicity was markedly reduced following spike ablation, corroborating the essential role of spike morphology in inducing lysosomal membrane damage (Figure S4G, Supporting Information). Notably, temperature tracking during laser treatment confirmed that no significant temperature changes occurred, ruling out heat‐related effects (Figure S4H, Supporting Information).

To further validate the mechanistic basis of spike‐induced lysosomal disruption, EGFP‐Gal3 plasmids were transfected into LLC cells. Fluorescence microscopy showed extensive Gal3 puncta formation with colocalization to lysosomes (85.2%) following 24‐h incubation with LSPs, which was indicative of LMP. In contrast, the colocalization efficiency of Gal3 with lysosomes was reduced in MSP‐ and SSP‐treated cells (55.9%, 42.8%), highlighting the dependency of lysosomal damage on the spike length (Figure 2G,H; Figure S5A, Supporting Information). Importantly, when laser ablation was applied 4 h post‐incubation with LSPs, Gal3 puncta were not observed, suggesting that early spike ablation prevented lysosomal damage (Figure 2G,H; Figure S5B, Supporting Information). Cell viability assays further confirmed that ablation significantly attenuated LSPs‐induced cytotoxicity (Figure S5C, Supporting Information). Collectively, these findings demonstrate a strong correlation between spike morphology and mechanical lysosomal disruption, offering critical insights for the rational design of precision nanotherapeutics targeting intracellular organelles.

### 2.3 Morphology‐Dependent Nanospikes Reveal Lysosomal Damage Stress Threshold

To further elucidate the mechanical interaction between nanospikes and lysosomal membranes, a comprehensive finite element simulation was conducted to model the physical behavior of nanospikes in contact with lysosomes. Based on established mechanical modeling frameworks, four nanoparticle variants were constructed, representing LSPs, MSPs, SSPs, and ablated spike geometries, with uniformly distributed spike arrays over their surfaces. Using a custom MATLAB algorithm, a uniform, non‐overlapping distribution of spike bases on the spherical surface was achieved via golden‐angle spiral placement combined with a binary search optimization. The final spatial coordinates were then imported into ABAQUS through Python scripting, enabling high‐fidelity 3D model reconstruction (Figure S6A,B, Supporting Information). These computational models were designed to replicate the experimentally observed spike dimensions and distributions, and uniform radial displacement boundary conditions were applied at the spike‐lipid membrane interface to simulate mechanical interaction.

**Figure 3:**
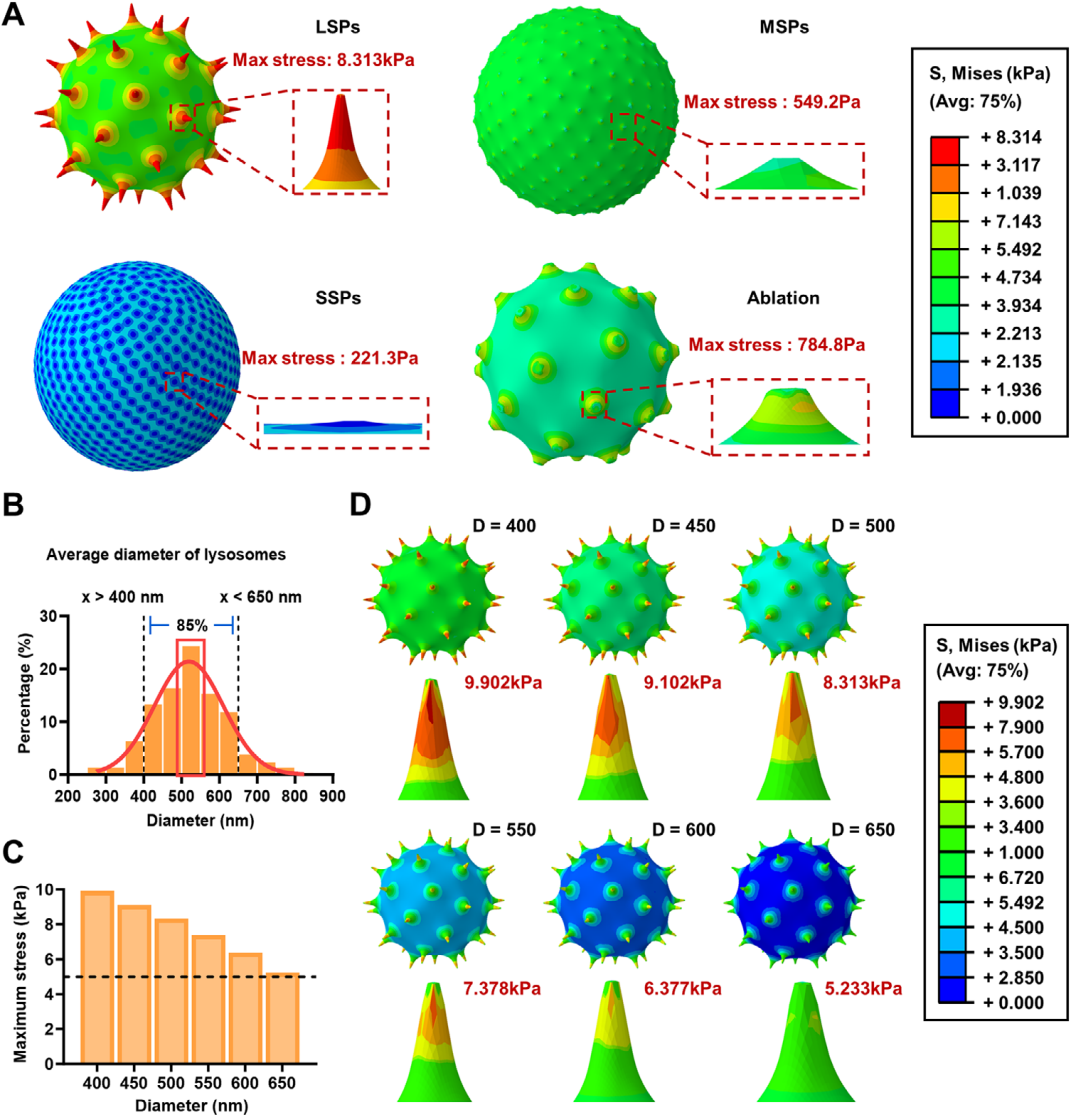
Finite element simulations reveal that nanospikes generate spike length‐dependent membrane stress on lysosomal membranes. A) Simulated von Mises stress distributions of lysosomes interacting with particles of different spike morphologies, including LSPs, MSPs, SSPs, and LSP plus laser ablation. B) Histogram of lysosome diameters in LLC cells determined by confocal imaging. C) Maximum stress values generated by LSPs across lysosomes of varying diameters. D) Simulated stress profiles of LSPs interacting with lysosomes of different diameters (400–650 nm).

To evaluate the stress distribution in lysosomal membranes resulting from spike‐tip forces, a spherical membrane model mimicking lysosomes with an average diameter of 500 nm was employed, and the maximum von Mises stress at the spike‐membrane contact interface was calculated. Notably, LSPs were found to generate a maximum stress of 8.313 kPa at the spike‐membrane contact points, which was 15.1‐fold to 37.6‐fold higher compared to MSPs and SSPs (**Figure** **3**A). These stress magnitudes differed by orders of magnitude, with the stress generated by LSPs being consistent with previously reported mechanical thresholds required for LMP.^[^ ^28^, ^29^ ^]^ Furthermore, nanosecond pulsed‐laser ablation of the LSPs was shown to significantly reduce spike length, and the mechanical stress decreased to 784.8 Pa (Figure 3A). Post‐ablation spike models exhibited markedly diminished stress values, confirming that the magnitude of membrane stress was directly dependent on spike sharpness and morphology.

Additional simulations of lysosome models with varying diameters within the 400–650 nm range were performed to evaluate the robustness of the observed trends (Figure 3B). Maximum stress levels remained within the 5.233–9.902 kPa range across the majority of lysosome sizes, particularly for LSPs, thereby reinforcing the hypothesis that the mechanical force exerted by the spikes was sufficient to compromise lysosomal membrane integrity in most cases (Figure 3C,D). Taken together, these simulations provide a quantitative framework to explain the experimental observations of lysosomal disruption and cytotoxicity. It was demonstrated that long nanospikes could exert mechanical forces that generated membrane stresses exceeding the critical damage threshold of lysosomal membranes, while laser ablation could effectively mitigate these stresses by altering the spike morphology. These findings support the hypothesis that lysosomal membrane damage induced by mechanical interaction with LSPs serves as a key initiating event in the cascade leading to cancer cell death.

### 2.4 LSPs Induce Autophagic Cell Death via the Gal3‐Trim16 Signaling Axis

**Figure 4:**
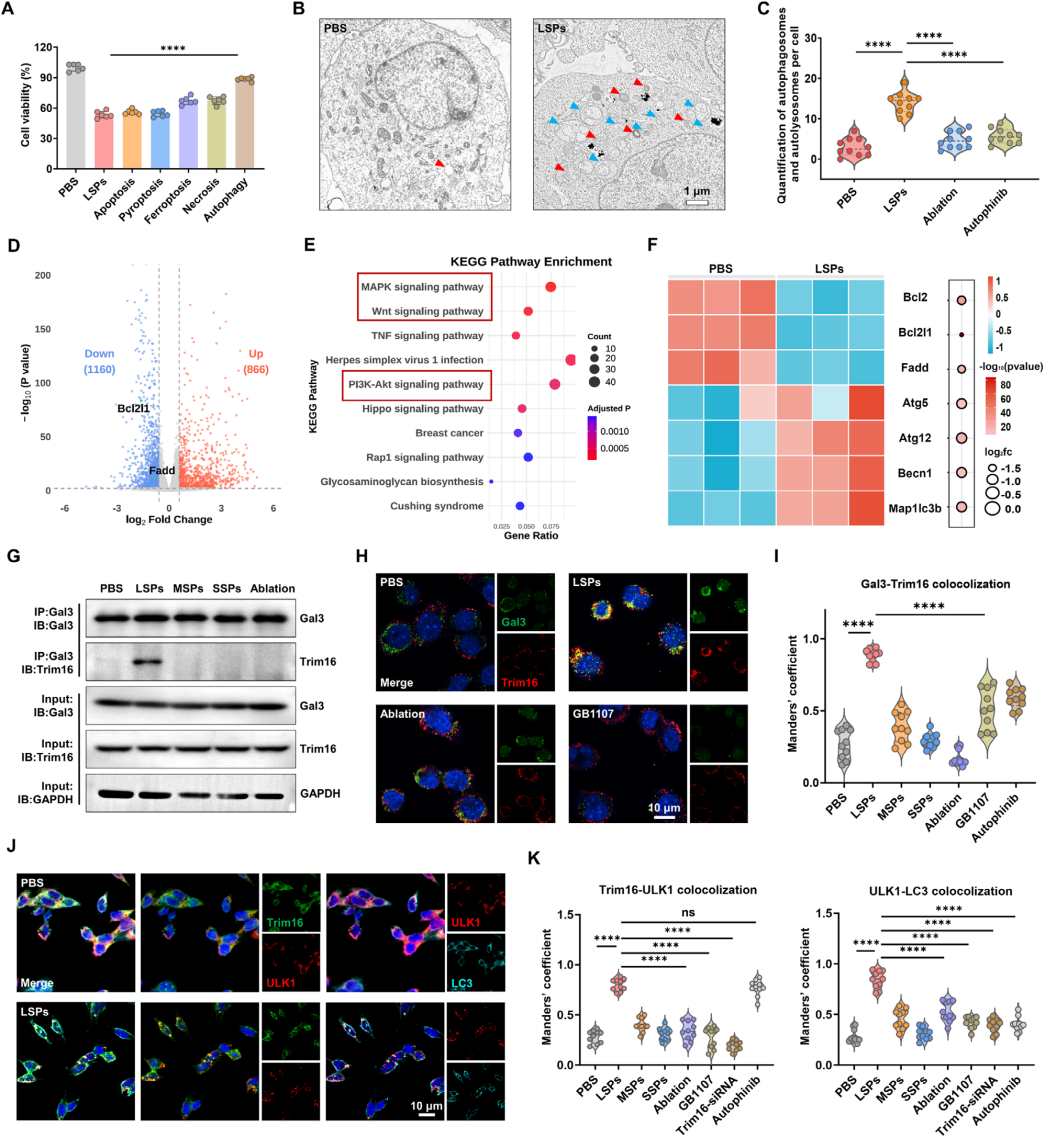
LSPs induce autophagic cell death via the Gal3‐Trim16 signaling axis. A) Cell viability of LLC cells after treatment with LSPs (32.77 µg mL^−1^) for 24 h or different cell death pathway inhibitors. Data are presented as mean ± s.d. Statistical significance: *p* < 0.05 (^*^), *p* < 0.01 (^**^), *p* < 0.001 (^***^), *p* < 0.0001 (^****^). B,C) Bio‐TEM images (B) and quantification of autophagosomes and autolysosomes (C) of LLC cells treated with LSPs (32.77 µg mL^−1^), LSPs plus laser ablation, or Autophinib. Red arrows indicate autolysosomes, and blue arrows indicate autophagosomes. Data are presented as mean ± s.d. Statistical significance: *p* < 0.05 (^*^), *p* < 0.01 (^**^), *p* < 0.001 (^***^), *p* < 0.0001 (^****^). D) Volcano plot of differentially expressed genes in LLC cells under LSPs versus PBS treatment. E) KEGG pathway enrichment analysis of downregulated signaling pathways after LSPs treatment. F) Heatmap of representative genes related to apoptosis and autophagy in PBS‐ and LSPs‐treated cells. G) Co‐IP analysis of Gal3‐Trim16 interaction in PBS, LSPs, MSPs, SSPs, and LSP plus laser ablation groups. H,I) Immunofluorescence staining of Gal3 (green), Trim16 (red), and Hoechst (blue) (H) and quantification by Manders’ coefficient of Gal3‐Trim16 colocalization efficiency (I) in LLC cells treated with LSPs, LSPs plus laser ablation, GB1107, or Autophinib. Data are presented as mean ± s.d. Statistical significance: *p* < 0.05 (^*^), p < 0.01 (^**^), *p* < 0.001 (^***^), *p* < 0.0001 (^****^). J,K) Immunofluorescence staining of Trim16 (green), ULK1 (red), and LC3 (cyan) (J) and quantification by Manders’ coefficient of Trim16‐ULK1 colocalization, as well as ULK1‐LC3 colocalization efficiency (K) in LLC cells treated with LSPs, MSPs, SSPs, LSPs plus laser ablation, GB1107, Trim16‐siRNA or Autophinib. Data are presented as mean ± s.d. Statistical significance: *p* < 0.05 (^*^), *p* < 0.01 (^**^), *p* < 0.001 (^***^), *p* < 0.0001 (^****^).

To further investigate the biological mechanism underlying nanospike‐induced cancer cell death, a comprehensive series of assays was conducted to evaluate the downstream consequences of lysosomal damage. Extensive and irreversible lysosomal damage has previously been reported to initiate regulated cell death pathways.^[^ ^30^, ^31^ ^]^ In accordance with this, different cell death pathway inhibitors were applied. The results showed a marked reduction in cytotoxicity upon treatment with autophagy inhibitors in LSPs‐treated cancer cells, suggesting that lysosomal damage primarily activated autophagic cell death mechanisms (**Figure** **4**A). Quantification of autophagy‐associated signaling pathways confirmed that autophagic activity was significantly elevated following treatment with LSPs in comparison to MSPs and SSPs (Figure S7A, Supporting Information). In addition, quantitative analysis of autophagosomes from bio‐TEM images further demonstrated that cells co‐incubated with LSPs significantly promoted autophagosome formation (Figure 4B,C, Figure S7B, Supporting Information). To substantiate the role of LSPs‐induced lysosomal damage in promoting autophagy‐mediated cell death, RNA sequencing and KEGG pathway enrichment analyses were performed. Transcriptomic profiling revealed substantial transcriptional reprogramming in LSPs‐treated LLC cells, characterized by a pronounced upregulation of autophagy‐associated genes and suppression of pathways related to cell proliferation, metabolism, and survival, including PI3K‐Akt, MAPK, and NF‐κB signaling pathways (Figure 4D,E; Figure S7C, Supporting Information). These observations suggested that autophagic dysregulation was a downstream consequence of mechanical lysosomal injury. Heatmap analysis showed elevated expression from the *Bcl‐2* (B‐cell lymphoma 2) and autophagy‐related gene (ATG) families, including *Becn1*, *Atg5*, *Atg12*, and *Trim16* genes, in LSPs‐treated cells (Figure 4F). The above results indicate that LSPs‐induced lysosomal damage activates autophagy‐related pathways, assuming that the increased expression of related genes directly contributes to autophagic cell death.

Based on the observed extensive recruitment of Gal3 upon LSPs‐mediated lysosomal disruption (Figure 2G,H) and the reported role of the Gal3‐Trim16 axis in initiating autophagic signaling,^[^ ^32^, ^33^ ^]^ additional investigations were performed to clarify its contribution to LSPs‐mediated cell death. Co‐immunoprecipitation (Co‐IP) assays validated the physical interaction between Gal3 and Trim16, with strong binding observed in the LSPs‐treated group but minimal or undetectable interaction in the PBS, MSPs, and SSPs groups (Figure 4G). Immunofluorescence analysis demonstrated significant Gal3 puncta formation and colocalization with Trim16 following 24‐h LSPs incubation, suggesting spike‐length‐dependent recruitment of Trim16 to damaged lysosomal membranes (Figure 4H,I; Figure S7D, Supporting Information). Notably, this recruitment was markedly reduced after nanosecond pulsed laser ablation of LSPs, underscoring the importance of spike structural integrity in triggering downstream responses.

To functionally validate the role of the Gal3‐Trim16 axis in mediating autophagic cell death, chemical inhibitors targeting Gal3 (GB1107) and autophagy (Autophinib) were applied. These inhibitors not only attenuated Gal3‐Trim16 colocalization and Trim16 puncta formation in immunofluorescence assays (Figure 4H,I; Figure S7D, Supporting Information), but also significantly improved cell viability in cytotoxicity assays and live/dead staining analyses (Figure S7E–G, Supporting Information). Furthermore, immunofluorescence colocalization of Trim16 with its downstream effector ULK1 (a canonical autophagy initiator), together with LC3 staining, revealed that LSPs markedly enhanced Trim16‐ULK1 colocalization and promoted LC3 expression (Figure 4J,K; Figure S7H,I, Supporting Information). Importantly, inhibition of Gal3 by GB1107 or silencing of Trim16 by related siRNA markedly reduced Trim16‐ULK1 and ULK1‐LC3 colocalization, highlighting the essential role of the Gal3‐Trim16‐ULK1 axis in LSPs‐induced autophagy‐dependent cell death. Notably, treatment with Autophinib did not affect Trim16‐ULK1 colocalization but reduced ULK1‐LC3 colocalization, indicating that the Gal3‐Trim16‐ULK1 axis could function as an upstream signaling cascade regulating LSP‐mediated autophagy (Figure 4J,K; Figure S7H,I, Supporting Information). Collectively, these results delineate a mechanistic pathway in which LSPs‐mediated mechanical disruption of lysosomes activates Gal3 recruitment and subsequent Trim16/ULK1 engagement, leading to excessive autophagy and cancer cell death.

### 2.5 LSPs Induce Autophagic Cell Death and Inhibit Tumor Growth In Vivo

**Figure 5:**
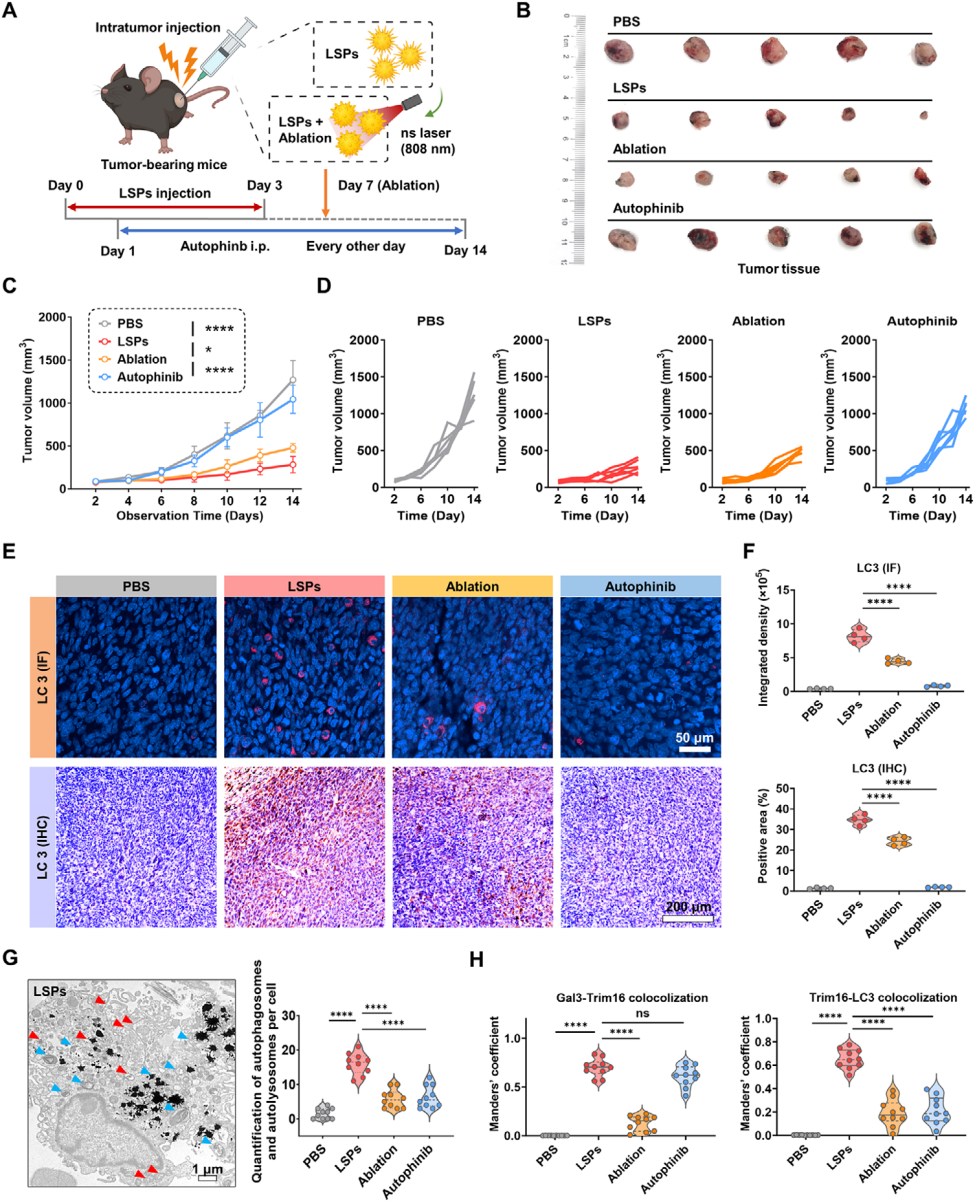
LSPs exhibit potent anticancer efficacy in vivo, which can be terminated by pulsed laser ablation. A) Schematic of the in vivo treatment protocol involving intratumoral injection of LSPs, LSPs plus laser ablation, or Autophinib administration. B) Representative tumor images from mice treated with PBS, LSPs, LSPs plus laser ablation, or Autophinib for 14 days. C,D) Tumor volume curves (C) and individual tumor growth trajectories (D) of mice treated with PBS, LSPs, LSPs plus laser ablation, or Autophinib for 14 days. Data are presented as mean ± s.d. Statistical significance: *p* < 0.05 (^*^), *p* < 0.01 (^**^), *p* < 0.001 (^***^), *p* < 0.0001 (^****^). E,F) LC3 immunofluorescence (IF, top) and immunohistochemistry (IHC, bottom) staining of tumor sections from mice treated with PBS, LSPs, LSPs plus laser ablation, or Autophinib for 14 days (E), and corresponding quantification of LC3 integrated density and positive area (F). Data are presented as mean ± s.d. Statistical significance: *p* < 0.05 (^*^), *p* < 0.01 (^**^), *p* < 0.001 (^***^), *p* < 0.0001 (^****^). G) Bio‐TEM images and quantification of autophagosomes and autolysosomes of LLC tumor tissues treated with LSPs, LSPs plus laser ablation, or Autophinib. Red arrows indicate autolysosomes, and blue arrows indicate autophagosomes. Data are presented as mean ± s.d. Statistical significance: *p* < 0.05 (^*^), *p* < 0.01 (^**^), *p* < 0.001 (^***^), *p* < 0.0001 (^****^). H) Quantification by Manders’ coefficient of Gal3‐Trim16 and Trim16‐LC3 colocalization efficiency in tumor tissue sections treated with LSPs, LSPs plus laser ablation, or Autophinib based on immunofluorescence staining of Gal3 (red), Trim16 (green), and LC3 (purple). Data are presented as mean ± s.d. Statistical significance: *p* < 0.05 (^*^), *p* < 0.01 (^**^), *p* < 0.001 (^***^), *p* < 0.0001 (^****^).

To verify the in vivo anticancer efficacy of LSPs, an LLC subcutaneous cancer model was established, and LSPs were intratumorally administrated three times, followed by a 14‐day observation period. (**Figure** **5**A). First, we demonstrated a concentration‐dependent cancer suppression effect, with higher LSPs dosages (400 µg) leading to more pronounced inhibition of tumor growth (Figure S8A,B, Supporting Information). To further regulate the biosafety of this treatment strategy, pulsed nanosecond laser ablation was introduced on day 7 to selectively terminate LSPs activity in vivo. Laser penetration testing revealed that sufficient energy could reach the tumor site through intact skin, indicating the feasibility of non‐invasive ablation (Figure S8C, Supporting Information). To evaluate the mechanism of tumor suppression, pharmacological inhibition of autophagy was performed via intraperitoneal injection of Autophinib. Tumor volume analysis showed that autophagy inhibition significantly reduced the tumor‐suppressive effect of LSPs (tumor inhibition rate: 17.8% in the Autophinib group vs. 77.8% in the LSPs group), confirming the functional role of autophagic cell death in LSPs‐mediated tumor clearance. Additionally, the tumor in the ablation group partially resumed growth after laser treatment on day 7, further supporting that the LSPs‐induced cytotoxic effect could be effectively terminated in vivo (Figure 5B–D; Figure S8D,E, Supporting Information).

To evaluate changes in autophagy within tumor tissues, immunofluorescence and immunohistochemistry staining of LC3, a classical autophagy marker, showed significantly higher expression levels in the LSPs group, which were markedly diminished following laser ablation or autophagy inhibition, confirming that autophagy was the main mode of cell death after 14 days of LSPs treatment (Figure 5E,F). In addition, quantitative bio‐TEM analysis of autophagosomes in tumor tissues further demonstrated a significant increase in autophagosome abundance upon LSP treatment, which was substantially reduced following either laser ablation or treatment with Autophinib (Figure 5G; Figure S9A, Supporting Information). Moreover, immunofluorescence colocalization staining of Gal3, Trim16, and LC3 in tumor sections revealed that LSPs treatment could promote Gal3‐Trim16‐LC3 colocalization, whereas laser ablation markedly reduced the colocalization efficiency of both Gal3‐Trim16 and Trim16‐LC3 (Figure 5H; Figure S9B, Supporting Information). Notably, treatment with Autophinib selectively decreased the colocalization efficiency of Trim16 with LC3. Collectively, these findings substantiate Gal3‐Trim16 as a key molecular axis mediating LSP‐induced autophagy‐dependent cell death in vivo.

In addition, histological analysis of PB staining revealed that LSPs were predominantly retained within tumor tissues (Figure S10A, Supporting Information). Hematoxylin and eosin (H&E) images demonstrated extensive areas of tumor cell death in the LSPs and Ablation groups, but not in the Autophinib group (Figure S10B, Supporting Information). Notably, Caspase‐3 immunostaining did not exhibit significant changes across groups, indicating that the observed cell death was not mediated by apoptosis but instead by autophagic cell death (Figure S10C, Supporting Information). Furthermore, histological assessments of major organs (heart, liver, spleen, lung, kidney, and brain) following 14 days of treatment revealed no evident signs of acute or chronic pathological toxicity in any of the experimental groups, demonstrating the biosafety of LSPs‐based treatment with or without laser ablation (Figure S11A, Supporting Information). Taken together, these findings confirm that LSPs could potently induce autophagic cell death for anticancer therapy. In addition, laser ablation provides a controllable strategy to modulate nanospike morphology and further reduce LSPs‐induced cytotoxicity. This work establishes a precise mechanical nanomedicine framework, offering new insights into the rational design of force‐responsive nanotherapeutics for targeted cancer treatment.

## 3 Discussion

Mechanical signals are increasingly recognized as critical physical factors that regulate biochemical signaling and cellular activities.^[^ ^1^, ^34^ ^]^ While previous studies have demonstrated that applying high mechanical force at the extracellular level can modulate cellular functions,^[^ ^13^, ^35^ ^]^ the in vivo application of such extracellular mechanical regulation remains limited. To overcome this limitation, recent advances have highlighted the potential of targeting intracellular organelles, which serve as central sites for both biochemical and mechanical signaling, to achieve subcellular‐level mechanical modulation.^[^ ^7^, ^36^ ^]^ In this study, we designed gold nanospikes with tunable lengths using IO as the core. These nanospikes can be efficiently internalized by cancer cells and selectively accumulated within lysosomes. The sharp tips of the nanospikes exert localized mechanical forces on the lysosomal membrane, inducing membrane stress that leads to LMP and subsequent cancer cell death. This organelle‐targeted mechano‐therapeutic strategy offers a promising avenue to overcome current barriers in mechanically driven cancer treatment.

High mechanical stress at the kPa level has been reported to cause physical disruption of biomimetic membrane structures, as demonstrated by atomic force microscopy (AFM) and related techniques.^[^ ^28^, ^29^ ^]^ However, the effects of comparable mechanical stress on intracellular organelle membranes remain poorly understood, despite the critical role of organelle membrane homeostasis in determining cell fate. In our study, we designed nanospikes with tunable lengths ranging from 45.4 to 254.2 nm, which were able to induce different degrees of membrane damage similar to those reported in previous studies.^[^ ^37^ ^]^ This approach allowed us to delineate the correlation between spike geometry and lysosomal perturbation, thereby linking mechanical parameters with downstream tumor cell responses. Importantly, we also identified the mechanical stress threshold required to induce LMP. Finite element modeling revealed that LSPs with a length of 254.2 nm could exert mechanical stress up to 8.313 kPa on the lysosomal membrane within an average‐sized lysosome (≈500 nm in diameter) of LLC cells, leading to widespread structural disruption and subsequent cancer cell death.

Moreover, the mechanical stress exerted by MSPs and SSPs on lysosomal membranes was only 6.6% and 2.7% that of LSPs, respectively, confirming sub‐kPa level stress was insufficient to induce widespread lysosomal membrane disruption. Importantly, utilizing nanosecond‐pulsed laser ablation significantly reduced the mechanical stress applied by LSPs on lysosomal membranes by ≈10.6‐fold, thereby effectively terminating LMP induction and cancer cell death. Furthermore, by modulating the lysosome size, we confirmed that LMP induction by LSPs occurred within a mechanical stress range of 5.233–9.902 kPa across lysosomes of varying diameters. These findings provide clear design parameters for future studies aiming to achieve organelle‐level mechanical regulation. Notably, because lysosomes are confined within the intracellular space, there is currently a lack of direct measurement tools to quantify the mechanical forces acting on their membranes. Moreover, although mechanosensitive fluorescent probes may provide a potential approach for characterizing lysosomal stress,^[^ ^38^ ^]^ their targeting specificity and sensitivity remain challenging, which could be further addressed in future work.

The use of spiky nanostructures to enhance cellular internalization and trigger LMP for anticancer effects represents an emerging frontier in nanomedicine. However, current research on spiky nanoparticles has primarily focused on their drug‐loading capabilities^[^ ^22^, ^23^ ^]^ and synergistic therapeutic strategies.^[^ ^24^, ^25^, ^26^ ^]^ In contrast, limited attention has been given to the intrinsic role of the spiky morphology itself. In particular, how sharp nanostructures elevate lysosomal mechanical stress to activate specific signaling pathways leading to cancer cell death remains poorly understood. In this study, we delineated the biochemical signaling mechanism underlying nanospike‐induced, length‐dependent cytotoxicity in cancer cells. Our results demonstrate that LSPs induce widespread LMP, which subsequently activates the Galectin‐3 (Gal3)‐Trim16 signaling axis to trigger autophagic cell death in cancer cells—independent of classical apoptotic pathways. Nevertheless, lysosomes act as critical signaling hubs, and their disruption can also activate additional cell death programs,^[^ ^39^, ^40^ ^]^ as supported by the results in Figure 4A. We speculate that both the form and extent of lysosomal damage may influence the pathways of cell death, with the pronounced lysosomal membrane disruption caused by LSPs favoring the induction of extensive autophagy. Overall, this strategy provides definitive mechanistic insight into subcellular mechanical signal transduction and its impact on cellular fate, offering a promising therapeutic avenue for apoptosis‐resistant cancer and other treatment‐refractory malignancies.

In summary, we constructed nanospikes with three distinct spike lengths that are capable of efficiently accumulating within cancer cell lysosomes. Furthermore, we defined the mechanical stress threshold required to induce widespread LMP. This mechanical damage subsequently activates autophagic cell death via the Gal3‐Trim16 signaling axis. Both pulsed‐laser ablation of LSPs and pharmacological inhibition of autophagy using Autophinib effectively abolished the cytotoxic effects of LSPs, highlighting the interdependent relationship between LMP induction and autophagic cell death. Importantly, our strategy demonstrated enhanced antitumor efficacy in vivo, with LSPs mediating a tumor inhibition rate of 77.8%. These findings underscore the unique potential of nanospikes in mechanically regulating intracellular organelles for anticancer therapy. Unlike conventional nanotherapeutics that rely on chemical payloads or ligand‐receptor interactions, our nanospikes functionalized as an effective class of mechanical drugs that could directly exert mechanical forces on the lysosomal membrane, inducing high membrane stress that triggers LMP and cancer cell death. Furthermore, by rationally engineering the morphology of nanospikes (e.g., spike length and density), it is possible to achieve potent anticancer efficacy at lower drug concentrations. Moving forward, as our understanding of intracellular mechanical signaling continues to evolve, mechanically active nanomedicines may represent a new frontier in the development of next‐generation cancer therapies.

## 4 Experimental Section

### Synthesis of Nanospikes

Zinc‐doped Fe_3_O_4_ nanoparticles (Zn_0.4_Fe_2.6_O_4_, ≈20 nm) were synthesized by thermal decomposition as previously reported. Ferric and zinc acetylacetonates (282.5 mg, 316.3 mg), tetraphenylbenzoic acid (400 mg), oleic acid (1.067 g), and dibenzyl ether (10.43 g) were mixed, degassed with Ar, and heated at 290 °C for 30 min. Nanoparticles were purified by ethanol and toluene washing. Gold nanospheres (GNSs, ≈10 nm) were prepared via citrate‐mediated hydrothermal reduction of HAuCl_4_. A gold solution was boiled before sodium citrate addition. The resulting wine‐red solution was filtered and stored at 4 °C. IO‐GNS complexes were fabricated by anchoring GNSs onto IO via Au‐S bonding (1 mg mL^−1^ IO, 47 nm GNSs, stirred 12 h). Nanospikes were synthesized by Ag⁺‐assisted underpotential deposition and selective gold growth on IO‐GNS using HAuCl_4_ (0.5 mm), HCl (20 mm), AgNO_3_ (0.06 mm), and AA (1 mm). After 12 h stirring with HS‐PEG5000‐COOH (3 mg), particles were purified and redispersed in deionized water.

### Optimization of Spiky Structure Parameters

The IO:GNS ratio was varied (1:0.1 to 1:10) and the resulting IO‐GNS complexes were subjected to identical spike growth conditions. Additionally, single‐variable optimization was conducted by varying HAuCl_4_, HCl, AgNO_3_, or AA concentrations while keeping the others constant.

### Nanoparticle Characterization

DLS and zeta potential were measured using Zetasizer Nano‐ZS90 (Malvern Instruments) at 25 °C. UV–vis absorbance of GNSs was recorded at 512 nm and calculated using ε = 5 × 10^6^ M^−1^ cm^−1^.

TEM (JEM‐2100F, 200 kV) and SEM (Quanta FEG 250) were used for morphology imaging. Elemental composition and mapping were analyzed via TEM‐EDS.

### Cytotoxicity Evaluation (CCK‐8 Assay)

LLC cells (1 × 10^4^ cells per well) were seeded into 96‐well plates and treated with long, medium, or short‐spike nanospikes at 0–3200 µg mL^−1^ for 24 h. Cell viability was assessed using a CCK‐8 assay (450 nm readout).

### LDH Release Assay

LLC cells were treated with nanospikes at the IC_50_ concentration of LSPs. Experimental groups included blank, positive control, and nanoparticle groups. LDH release was measured from supernatants after 24 h using LDH cytotoxicity assay kit (C0016).

### ICP‐MS Cellular Uptake Analysis

LLC cells were incubated with LSPs (32.77 µg mL^−1^) for 0–48 h. After collection and aqua regia digestion, intracellular Au and Fe content was quantified by ICP‐MS. All nanospike concentrations used in this study were defined according to the elemental Au content.

### Cell Transfection and Confocal Imaging

LLC cells (1 × 10^5^) were seeded into 35 mm culture dishes and incubated with FITC‐labeled LSPs (32.77 µg mL^−1^) for 24 h. The cells were then stained with Hoechst (1:1000) and LysoTracker Red (1:1000), then followed by imaging using a confocal microscope (Leica TCS SP5).

For the Gal3‐LAMP1 colocalization assay, LLC cells were transfected with EGFP‐Gal3 plasmid using Jetprime (2.5 µg plasmid + 4 µL reagent) for 24–48 h. Cells were treated with LSPs, MSPs, SSPs and LSPs plus laser ablation (32.77 µg mL^−1^) for 24 h. Ablation was performed using nanosecond pulse laser (32 mJ) irradiation 4 h after the addition of LSPs. Then cells were fixed in 4% PFA, permeabilized with 0.2% Triton X‐100, blocked with 5% BSA for 1 h, and incubated overnight at 4 °C with primary antibodies (anti‐LAMP1, Abmart, 1:200). After PBS washes, sections were incubated with Alexa Fluor 555 conjugated secondary antibodies for 1 h at room temperature in the dark. Nuclei were counterstained with DAPI (1:1000), and images were captured using a confocal laser scanning microscope (Leica TCS SP5).

For the Trim16‐ULK1‐LC3 colocalization assay, LLC cells were treated with LSPs, MSPs, SSPs, LSPs plus laser ablation, GB1107 (1:1000, MCE), Trim16‐siRNA, Autophinib (1:1000, MCE). All nanospikes were co‐cultured with cells at the Au concentration of 32.77 µg mL^−1^ for 24 h. Then cells were fixed in 4% PFA, permeabilized with 0.2% Triton X‐100, blocked with 5% BSA for 1 h, and incubated overnight at 4 °C with primary antibodies (anti‐Trim16, Boster, 1:200; anti‐ULK1, Affinity, 1:200; anti‐LC3, Abmart, 1:200). After PBS washes, sections were incubated with Alexa Fluor‐conjugated secondary antibodies for 1 h at room temperature in the dark. Nuclei were counterstained with DAPI (1:1000), and images were captured using a confocal laser scanning microscope (Leica TCS SP5).

### Bio‐TEM Sample Preparation

LLC cells were incubated with LSPs (32.77 µg mL^−1^) for 24 h, fixed in 2% glutaraldehyde for 48 h, post‐fixed with osmium tetroxide, dehydrated in graded acetone, and embedded in EPON. Ultrathin sections (50 nm) were stained and imaged using TEM (JEM‐2100F, 200 kV).

### Geometric Reconstruction and Finite Element Setup

To generate a physically realistic distribution of nanospikes on spherical particles, a custom MATLAB algorithm was implemented that arranged non‐overlapping spike bases across the particle surface. The interface between each spike and the core particle was geometrically represented as a spherical cap with a half‐apex angle α, estimated from SEM images by quantifying the number of spikes along the particle perimeter in 2D projection. A golden‐angle spiral algorithm was used to uniformly distribute spike centers, and a binary search strategy was employed to determine the maximum number of spikes that could be placed without overlap, based on geometric intersection checks. The final spatial coordinates were exported and used to construct spike‐decorated nanoparticle models via Python scripting in ABAQUS.

The lysosomal membrane was modeled as a spherical shell discretized with four‐node reduced integration shell elements (S4R). Each spike was simplified as an outward‐pointing analytic rigid shell, omitting central core structure. In the initial configuration, all spikes were embedded just inside the membrane cavity. Their orientations were assigned based on spherical coordinates computed from MATLAB, which ensured uniform angular distribution and non‐overlapping placement. Contact between spikes and membrane was defined using a surface‐to‐surface formulation with the kinematic contact method.

### Lysosomal Membrane Material Model

The membrane was treated as an isotropic, nearly incompressible hyperelastic material, implemented using the Neo‐Hookean model consistent with prior studies of cell membrane mechanics. With an experimentally derived Young's modulus of 1000 Pa and Poisson's ratio of 0.4, the shear modulus was computed as G = 357.14 Pa, yielding material parameters C_10_ = 178.57 Pa and D_1_ = 0.0012 Pa^−1^, which were input directly into ABAQUS.

### Quasi‐static Spike‐Membrane Interaction Simulation

To approximate quasi‐static behavior while resolving complex contact deformations, an explicit dynamic solver was employed in ABAQUS. Prior to the main simulation, eigenvalue extraction was performed to determine the membrane's lowest natural frequency and derive a stable time increment. During simulation, kinetic energy was kept below 5% of internal energy to suppress dynamic effects.

To simulate the protrusion of the spikes, a slow outward displacement was imposed along each spike's radial direction—modifying only the radial component r of their spherical coordinate without changing the angular direction. This approach avoided numerical instabilities typically encountered when establishing contact, and enabled reliable stress distribution analysis in the final configuration.

### Cell Death Pathway Assay

LLC cells were seeded into 35mm cell culture dishes at a density of 1 × 10^5^ cells per well and incubated overnight. Then cells were co‐cultured with LSPs (32.77 µg mL^−1^) for 24h. To evaluate the contribution of different cell death pathways, the following inhibitors were added 4 h prior to analysis: z‐VAD‐FMK for apoptosis (10 µm, APExBIO), Necrostatin‐1 for necroptosis (20 µm, APExBIO), 3‐Methyladenine for autophagy (5 mm, APExBIO), Ferrostatin‐1 for ferroptosis (2 µm, Selleck), and MCC950 for pyroptosis (5 µm, MCE). After 4 h, cell viability was assessed using the CCK‐8 assay. Absorbance at 450 nm was measured using a microplate reader.

### RNA Sequencing

Total RNA was extracted using TRIzol, precipitated with isopropanol, and purified with 75% ethanol. After QC with NanoDrop 2000, mRNA was enriched by polyT selection and used to construct cDNA libraries. Libraries were quantified by Qubit and qPCR, then sequenced using Illumina NovaSeq 6000 (PE150) at Berry Genomics.

### Co‐Immunoprecipitation and Western Blotting

Cell lysates were incubated with 3 µg Gal3 antibody or IgG overnight at 4 °C, followed by 2 h incubation with protein A/G beads. After washing and SDS elution, eluates and input lysates were analyzed by SDS‐PAGE and immunoblotted with Gal3 and Trim16 antibodies. Chemiluminescence detection was performed, and band intensity was quantified using ImageJ.

### Anticancer Therapy In Vivo

All animal procedures were conducted in accordance with the institutional guidelines and approved by the Animal Care and Use Committee of Tongji University. C57BL/6 mice (male, 4 weeks old) were subcutaneously inoculated with 1 × 10^6^ LLC cells in the right groin region. Tumor volume was calculated using the formula: V (mm^3^) = 0.5 × width^2^ × length. When the tumor reached ≈50 mm^3^, mice were randomly assigned to four treatment groups (*n* = 5 per group): PBS, LSPs, LSPs plus laser ablation, and Autophinib. Mice received a single intratumoral injection of LSPs (20 mg kg^−1^, in 50 µL PBS) on Day 0. For the ablation group, tumors were exposed to nanosecond pulsed laser irradiation (808 nm, 32 mJ, 10 min) on Day 7 post‐injection. In the Autophinib group, mice were intraperitoneally administrated Autophinib (10 mg kg^−1^, MCE) every two days starting from Day 0. Tumor volume and body weight were monitored every other day. On Day 14, mice were euthanized and tumor were excised for imaging and histological analysis. Immunofluorescence (IF) and immunohistochemistry (IHC) staining for LC3 were performed on tumor sections to assess autophagy activation in each group.

### IF Staining

Tumor tissues were harvested and fixed in 4% paraformaldehyde at 4 °C overnight, followed by dehydration in 30% sucrose solution. Samples were embedded in OCT and sectioned at 5 µm thickness. Sections were permeabilized with 0.2% Triton X‐100, blocked with 5% BSA for 1 h, and incubated overnight at 4 °C with primary antibodies (e.g., anti‐Gal3, Proteinetch, 1:200; anti‐Trim16, Boster, 1:200; anti‐LC3, Abmart, 1:200). After PBS washes, sections were incubated with Alexa Fluor‐conjugated secondary antibodies for 1 h at room temperature in the dark. Nuclei were counterstained with DAPI (1:1000), and images were captured using a confocal laser scanning microscope (Leica TCS SP5).

### IHC Staining

Paraffin‐embedded tumor tissues were sectioned at 5 µm thickness, deparaffinized in xylene, and rehydrated through graded ethanol. Antigen retrieval was performed using citrate buffer (pH 6.0) at 95 °C for 15 min. Endogenous peroxidase activity was blocked with 3% hydrogen peroxide, followed by incubation with 5% BSA for 30 min. Sections were then incubated with primary antibodies (e.g., anti‐LC3, anti‐Caspase 3, Proteinetch, 1:100) overnight at 4 °C, followed by HRP‐conjugated secondary antibody for 1 h. DAB substrate was applied for signal development. Sections were counterstained with hematoxylin, dehydrated, and mounted for imaging under a bright‐field microscope.

### Biosafety Evaluation

C57BL/6 mice bearing LLC cells were treated with PBS, LSPs, LSPs plus laser ablation, and Autophinib. After 14 days, mice were sacrificed and major organs (heart, liver, spleen, lung, kidney, brain) were collected, fixed in 4% paraformaldehyde, and embedded in paraffin. Tissue sections (5 µm) were stained with hematoxylin and eosin (H&E) and imaged using a bright‐field microscope to assess histopathological changes.

### Statistics and Reproducibility

All graphs were performed using GraphPad Prism (v10) or R (v4.5.1). All data were repeated at least 3 times with similar outcomes and presented as mean ± s.d. ^*^ *p* < 0.05, ^**^ *p* < 0.01, ^***^ *p* < 0.001, ^****^ *p* < 0.0001.

## Conflict of Interest

The authors declare no conflict of interest.

## Author Contributions

Y.L., Z.G. and J.F. contributed equally to this work. Y.L., Z.G., and J.F. conceived this work. Y.L., Z.G., J.F., Y.T., C.C., and Y.C. designed the experiments. Y.L., Z.G., and R.Z. contributed to the synthesis and characterization of nanospikes. Y.L., Z.G., and J.F. performed the finite element simulations. Y.L., Z.G., R.Z., J.L., Z.H., W.G., M.Z., and C.X. contributed cell and animal experiments. Y.L., Z.G., and J.F. analyzed the data. Y.L., Z.G., and J.F. wrote the manuscript under the supervision of Y.T., C.C., and Y.C.

## Supporting information



Supporting Information

## Data Availability

The data that support the findings of this study are available from the corresponding author upon reasonable request.
