# Supplementary material for: Intracellular Mechanical Stress‐Mediated Autophagy Cell Death via Nanospikes for Cancer Treatment
Source: Adv Sci (Weinh). 2025 Oct 13;13(2):e12256. doi: 10.1002/advs.202512256 (PMC12786270; doi:10.1002/advs.202512256)
Supplement: Supplementary file 1 — Supporting Information [file ADVS-13-e12256-s001.docx]

**Intracellular Mechanical Stress-Mediated Autophagy Cell Death via Nanospikes for Cancer Treatment**

Yingze Li^1,2,3,4#^, Zihan Guo^1,2,3,4#^, Jiawei Fan^5#^, Ruimei Zhou^2,3,4^, Jiayan Li^6^, Zhixiang Hu^1,2,3,4^, Weicheng Gu^2,3,4^, Mengge Zheng^7^, Chang Xu^2,3,4^, Yichao Tang^5*^, Chang Chen^1*^, Yu Cheng^2,3,4*^

^1^Department of Thoracic Surgery, Shanghai Pulmonary Hospital, Tongji University School of Medicine, Shanghai, China.

^2^Translational Research Institute of Brain and Brain-Like Intelligence, Shanghai Fourth People’s Hospital, School of Medicine, Tongji University, Shanghai, China.

^3^State Key Laboratory of Autonomous Intelligent Unmanned Systems, Tongji University, Shanghai, China.

^4^Collaborative Innovation Center for Brain Science, Tongji University, Shanghai, China.

^5^School of Mechanical Engineering, Tongji University, Shanghai, China.

^6^Institute of Acoustics School of Physics Science and Engineering, Tongji University, Shanghai, China.

^7^Central Laboratory, Shanghai Pulmonary Hospital, Tongji University School of Medicine, Shanghai, China.

^#^These authors contributed equally.

^*^Correspondence: tangyichao@tongji.edu.cn (Y.T.); changchenc@tongji.edu.cn (C.C.); yucheng@tongji.edu.cn (Y.C.)


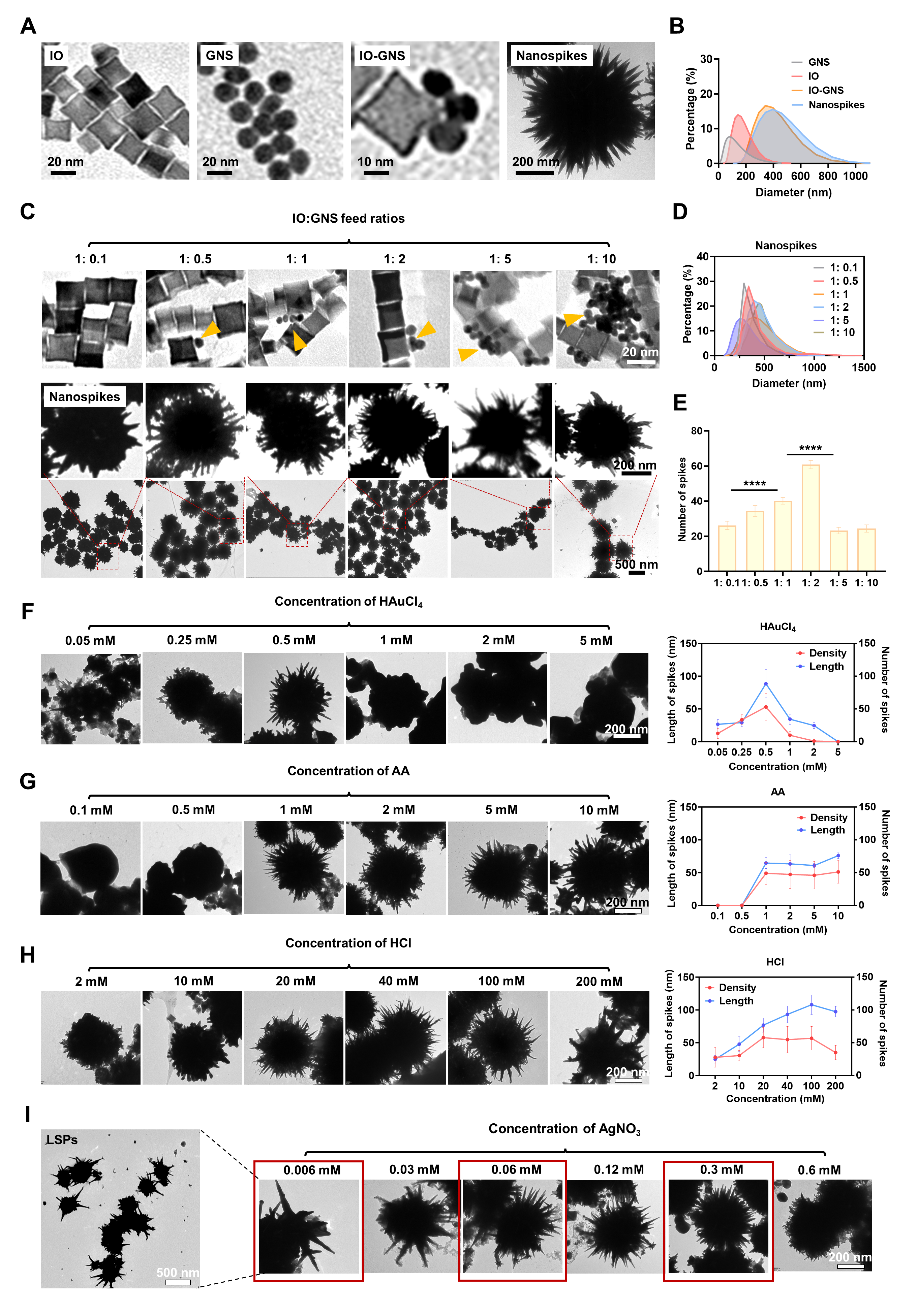


**Figure S1. Synthesis of nanospikes with different spike lengths.** A, B) TEM images (A) and hydrodynamic diameters (B) of IO, GNS, IO-GNS, and Nanospikes. C-E) TEM images (C), hydrodynamic diameters (D), and quantification of spike numbers (E) of nanospikes synthesized under different IO:GNS feed ratios. Data are presented as mean ± s.d. Statistical significance: p < 0.05 (*), p < 0.01 (**), p < 0.001 (***), p < 0.0001 (****). F-H) TEM images and quantification of spike numbers of nanospikes synthesized with varying concentrations of HAuCl₄ (F), AA (G), and HCl (H). (I) TEM images of nanospikes synthesized with varying concentrations AgNO₃.

**
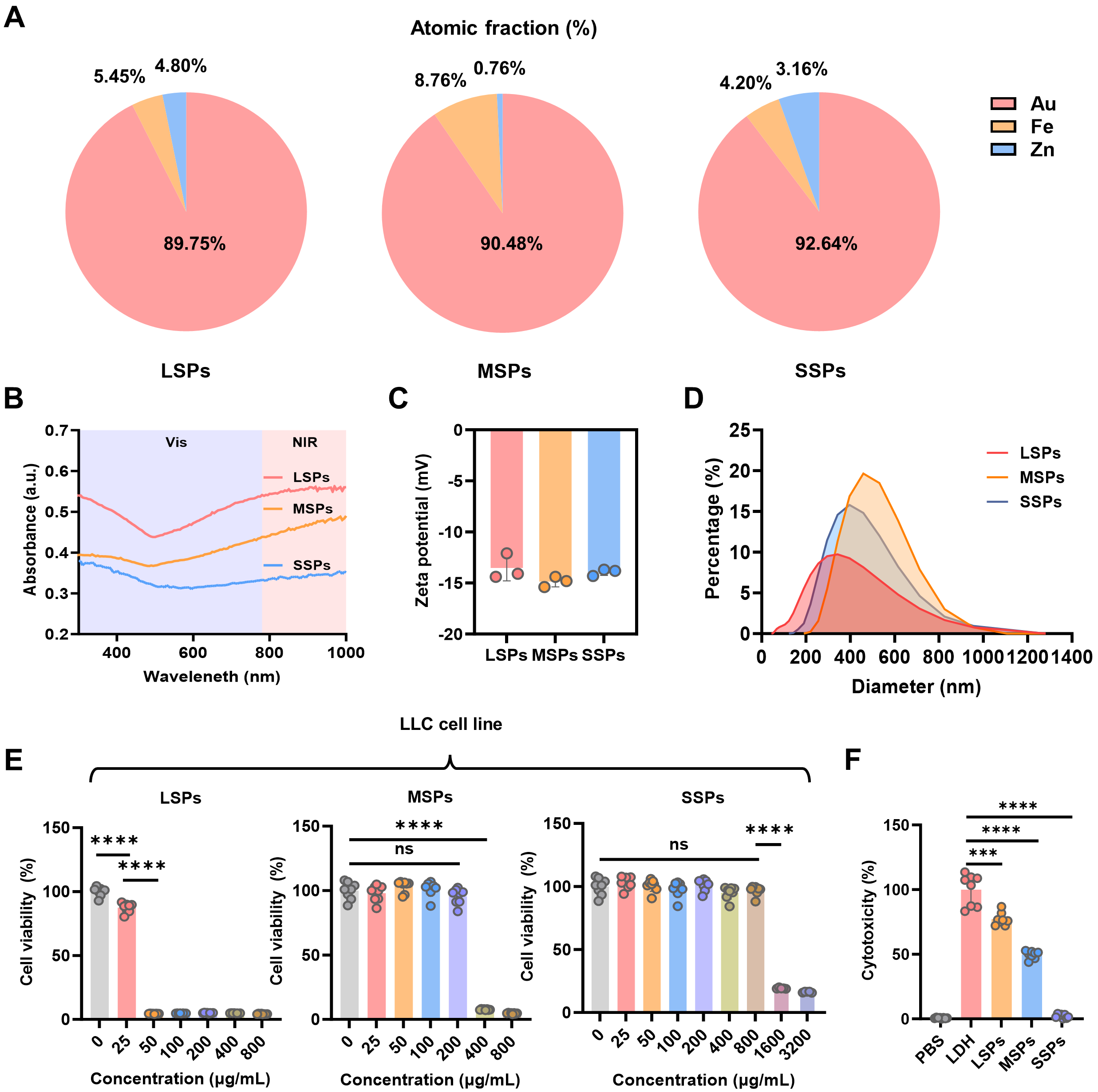
**

**Figure S2. Characterization of nanospikes with different spike lengths and the cytotoxicity toward cancer cells.** A) Elemental composition (Au, Fe, Zn) of long-spiked (LSPs), medium-spiked (MSPs), and short-spiked particles (SSPs) determined by elemental mapping. B–D) UV-vis absorbance spectra (B), zeta potential (C) and hydrodynamic diameters (D) of LSPs, MSPs, and SSPs. E) Cell viability of LLC after 24 h incubation with LSPs, MSPs, and SSPs at various Au concentrations. Data are presented as mean ± s.d. Statistical significance: p < 0.05 (*), p < 0.01 (**), p < 0.001 (***), p < 0.0001 (****). F) LDH release from LLC cells after 24 h incubation with LSPs, MSPs, and SSPs at the IC₅₀ concentration of LSPs (32.77 μg/mL). Data are presented as mean ± s.d. Statistical significance: p < 0.05 (*), p < 0.01 (**), p < 0.001 (***), p < 0.0001 (****).


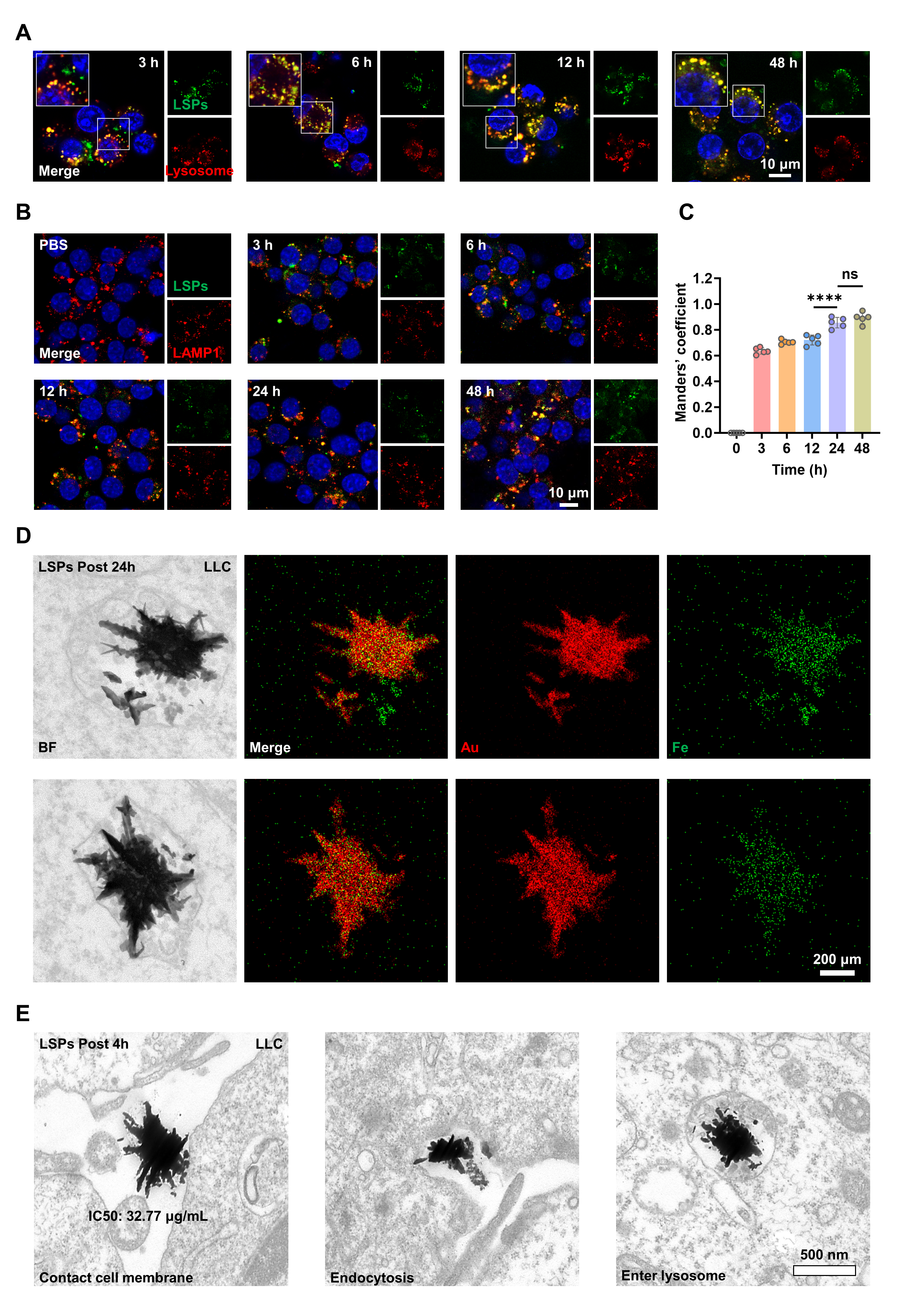


**Figure S3. Lysosomal localization of LSPs.** A) Confocal fluorescence imaging of LSPs (green) colocalized with lysosomes (red) in LLC cells for 3, 6, 12, or 48 h. Lysosomes were stained with LysoTracker Red (red), and nuclei were stained with Hoechst (blue). B, C) Confocal fluorescence imaging of LSPs (green) colocalized with lysosomes (red) in LLC cells. Lysosomes were stained with anti-LAMP1 antibody (red), and nuclei were stained with Hoechst (blue) (B). Quantification of colocalization at different time points using Manders’ coefficient (C). Data are presented as mean ± s.d. Statistical significance: p < 0.05 (*), p < 0.01 (**), p < 0.001 (***), p < 0.0001 (****). D) Elemental mapping images of gold (Au) and iron (Fe) in LSPs (32.77μg/mL) within LLC cells for 24 h. E) Subcellular distribution of LSPs (32.77 μg/mL) in lysosomes of LLC cells after co-culture for 4 h by bio-TEM.


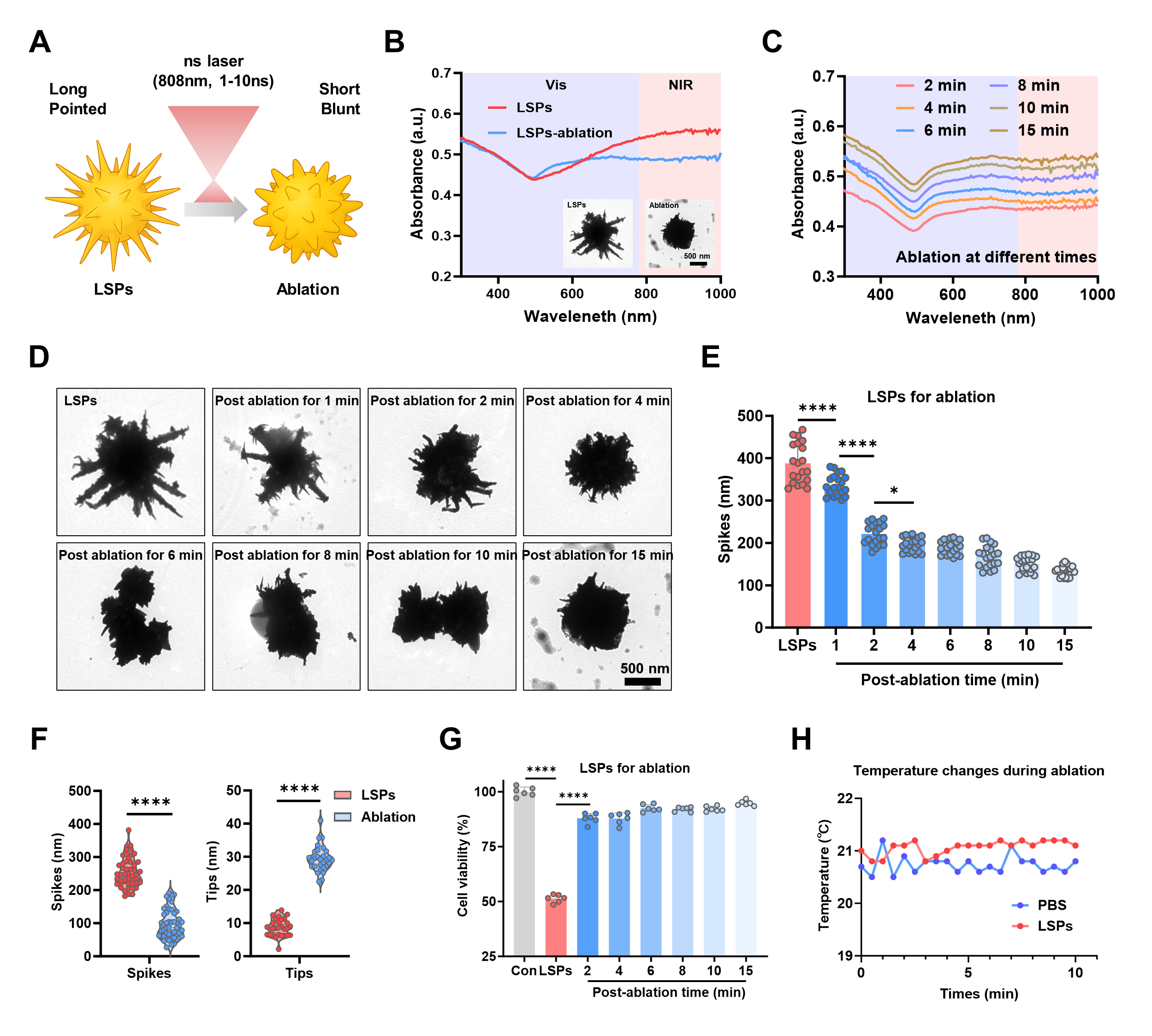


**Figure S4. Characterization of nanosecond pulsed laser-mediated ablation of LSPs.** A) Schematic illustration of nanosecond pulsed laser ablation. B, C) UV-vis absorbance spectra of LSPs before and after laser ablation (B), and after different ablation durations (C). D-F) TEM images (D), statistical analysis of spike length (E) of LSPs after different ablation times, and length of spikes and tips (F) of LSPs after ablation. Data are presented as mean ± s.d. Statistical significance: p < 0.05 (*), p < 0.01 (**), p < 0.001 (***), p < 0.0001 (****). G) Cytotoxicity of LSPs (32.77 μg/mL) with varying ablation durations toward LLC cells. Data are presented as mean ± s.d. Statistical significance: p < 0.05 (*), p < 0.01 (**), p < 0.001 (***), p < 0.0001 (****). H) Measurement of local temperature of cells and LSPs co-incubation following laser irradiation.

**
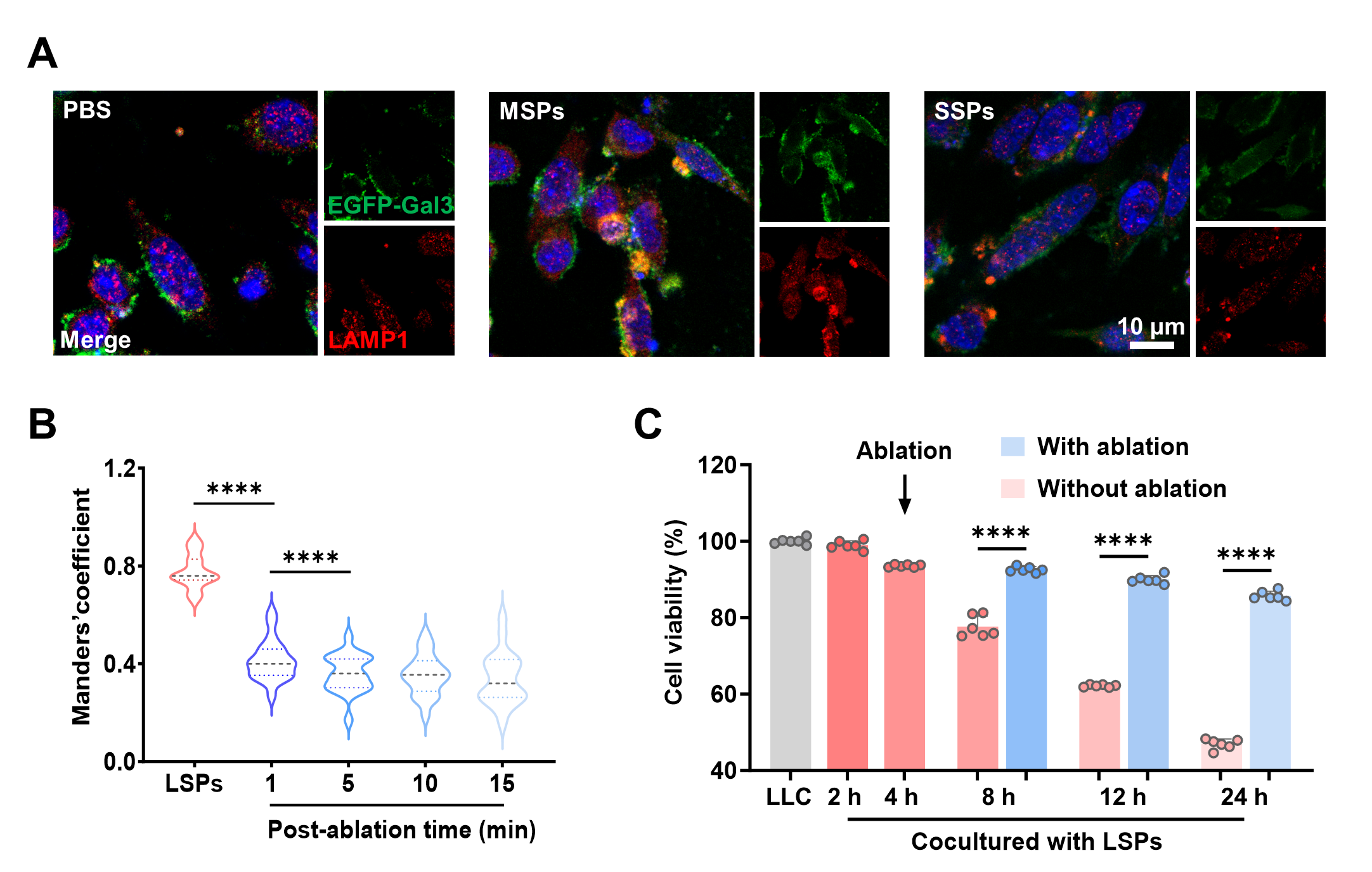
**

**Figure S5. Characterization of lysosomal damage and cytotoxicity induced by LSPs, MSPs, and SSPs.** A) Fluorescence images of EGFP-Gal3-transfected LLC cells treated with PBS, MSPs and SSPs for 24 h. Anti-LAMP1 antibody (red), and nuclei were stained with Hoechst (blue). B) Quantification of Gal3 and lysosomes colocalization efficiency using Manders’ coefficient in LLC cells incubated with LSPs (32.77 μg/mL) or laser-ablated LSPs for different durations. Data are presented as mean ± s.d. Statistical significance: p < 0.05 (*), p < 0.01 (**), p < 0.001 (***), p < 0.0001 (****). C) Cell viability of LLC cells after incubation with LSPs at the IC_50_ concentration (32.77 μg/mL) with or without laser ablation at various time points. Data are presented as mean ± s.d. Statistical significance: p < 0.05 (*), p < 0.01 (**), p < 0.001 (***), p < 0.0001 (****).

**
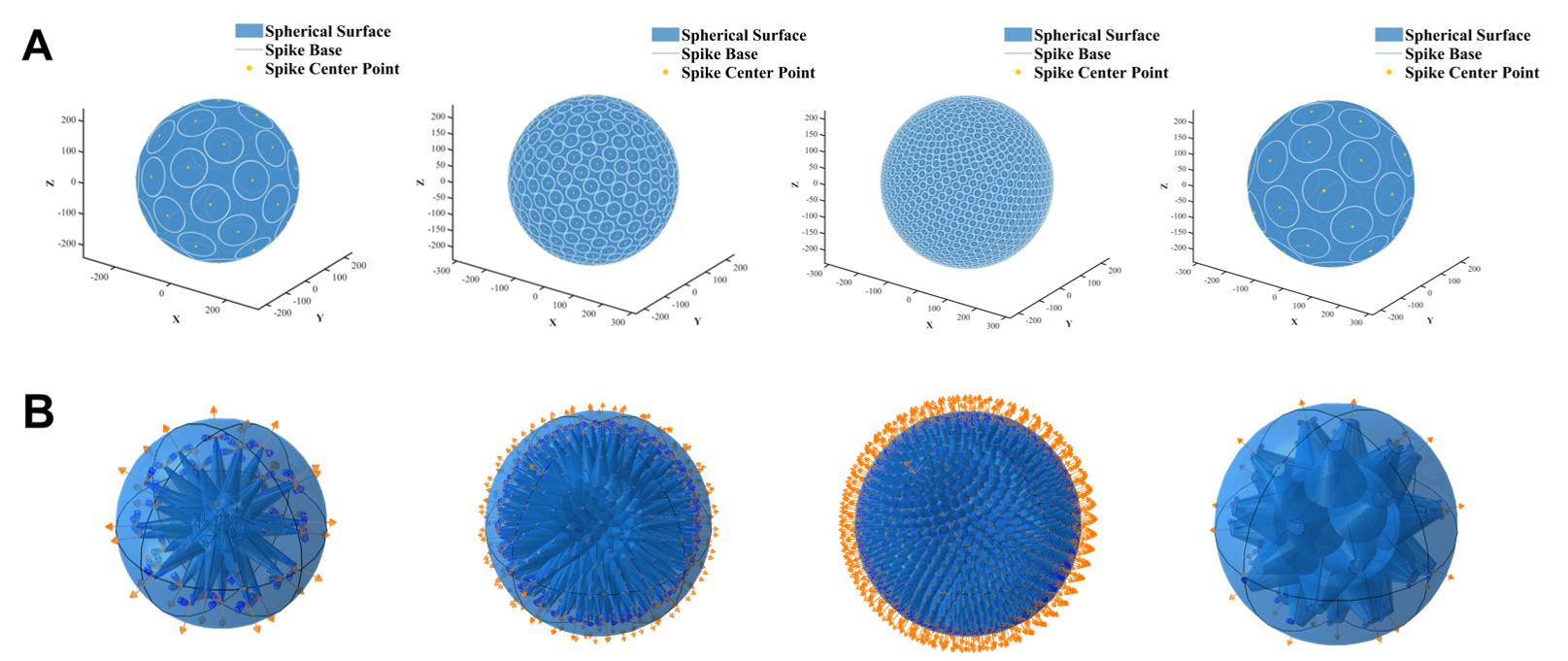
**

**Figure S6. Geometric reconstruction and boundary condition setup for finite element simulation of nanospikes.** A) Geometric reconstruction of spherical particles with varied spike densities for simulating mechanical interaction. B) Assignment of boundary conditions in ABAQUS for simulating four nanoparticle variants, used to assess spike-induced membrane stress.

**
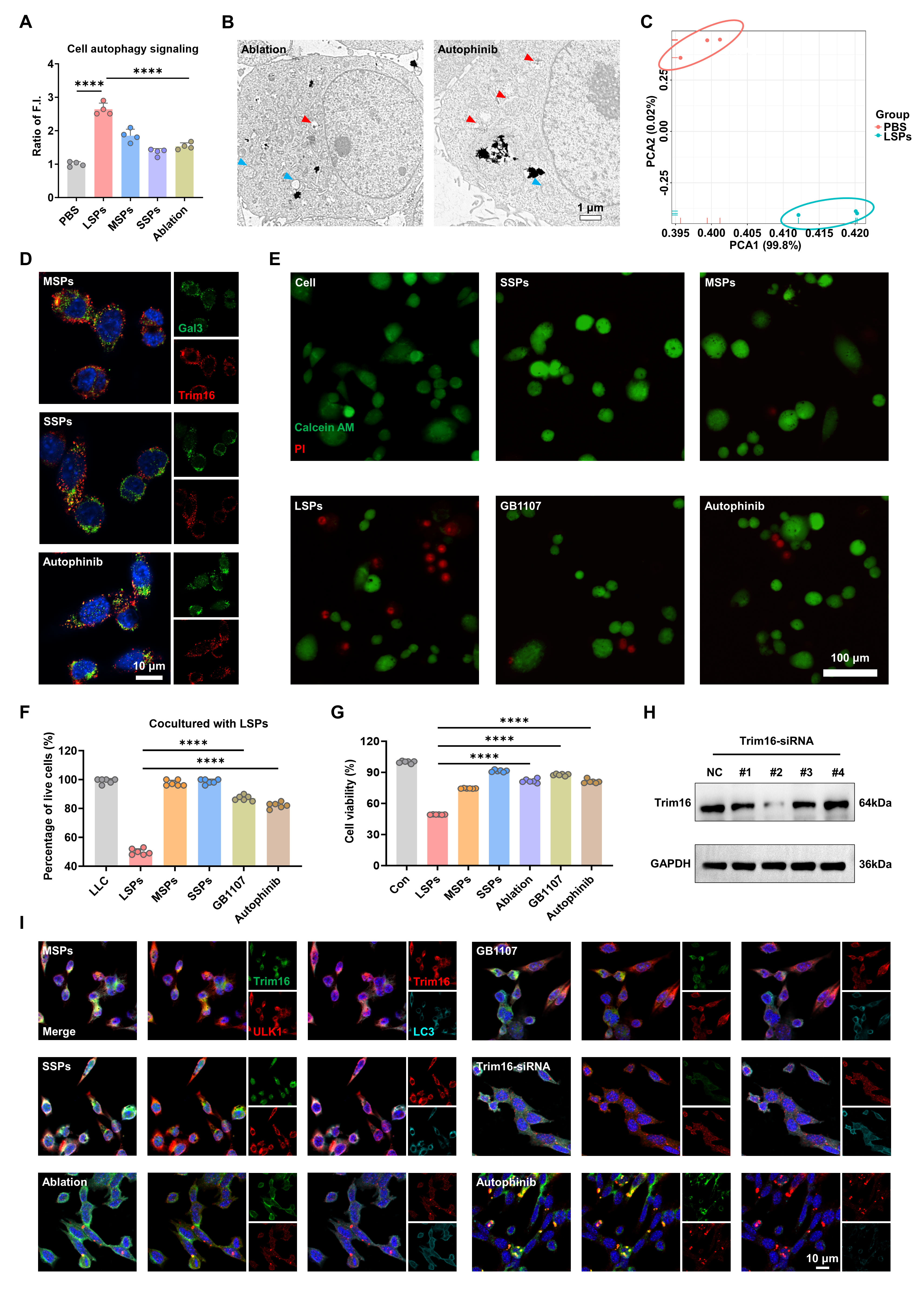
**

**Figure S7. Evaluation of Gal3-Trim16-ULK1 signaling in LLC cells mediated by LSPs.** A) Quantification of autophagy staining after treatment with LSPs, MSPs, SSPs and LSP plus laser ablation (32.77 μg/mL). Data are presented as mean ± s.d. Statistical significance: p < 0.05 (*), p < 0.01 (**), p < 0.001 (***), p < 0.0001 (****). B) Bio-TEM images of LLC cells treated with LSPs, LSPs plus laser ablation, or Autophinib. Red arrows indicate autolysosomes, and blue arrows indicate autophagosomes. C) Principal component analysis (PCA) of transcriptomic profiles from PBS- and LSP-treated LLC cells. D) Fluorescence images and quantification of cell viability in LLC cells co-incubated with MSPs, SSPs, and Autophinib. E, F) Live/dead staining of LLC cells under LSPs (32.77 μg/mL), LSP plus laser ablation, GB1107, or Autophinib treatment (E), and quantification of percentage of line cells (F). Data are presented as mean ± s.d. Statistical significance: p < 0.05 (*), p < 0.01 (**), p < 0.001 (***), p < 0.0001 (****). G) Cell viability of LLC cells under LSPs, MSPs, SSPs, LSP plus laser ablation, GB1107, or Autophinib treatment. Data are presented as mean ± s.d. Statistical significance: p < 0.05 (*), p < 0.01 (**), p < 0.001 (***), p < 0.0001 (****). H) Western blot results of Trim16 in LLC cells. Cells were transfected with either NC-siRNA or Trim16-siRNAs (#1, #2, #3, #4). I) Immunofluorescence staining of Trim16 (green), ULK1 (red), and LC3 (cyan) in LLC cells treated with LSPs, MSPs, SSPs, LSPs plus laser ablation, GB1107, Autophinib or Trim16-siRNA.


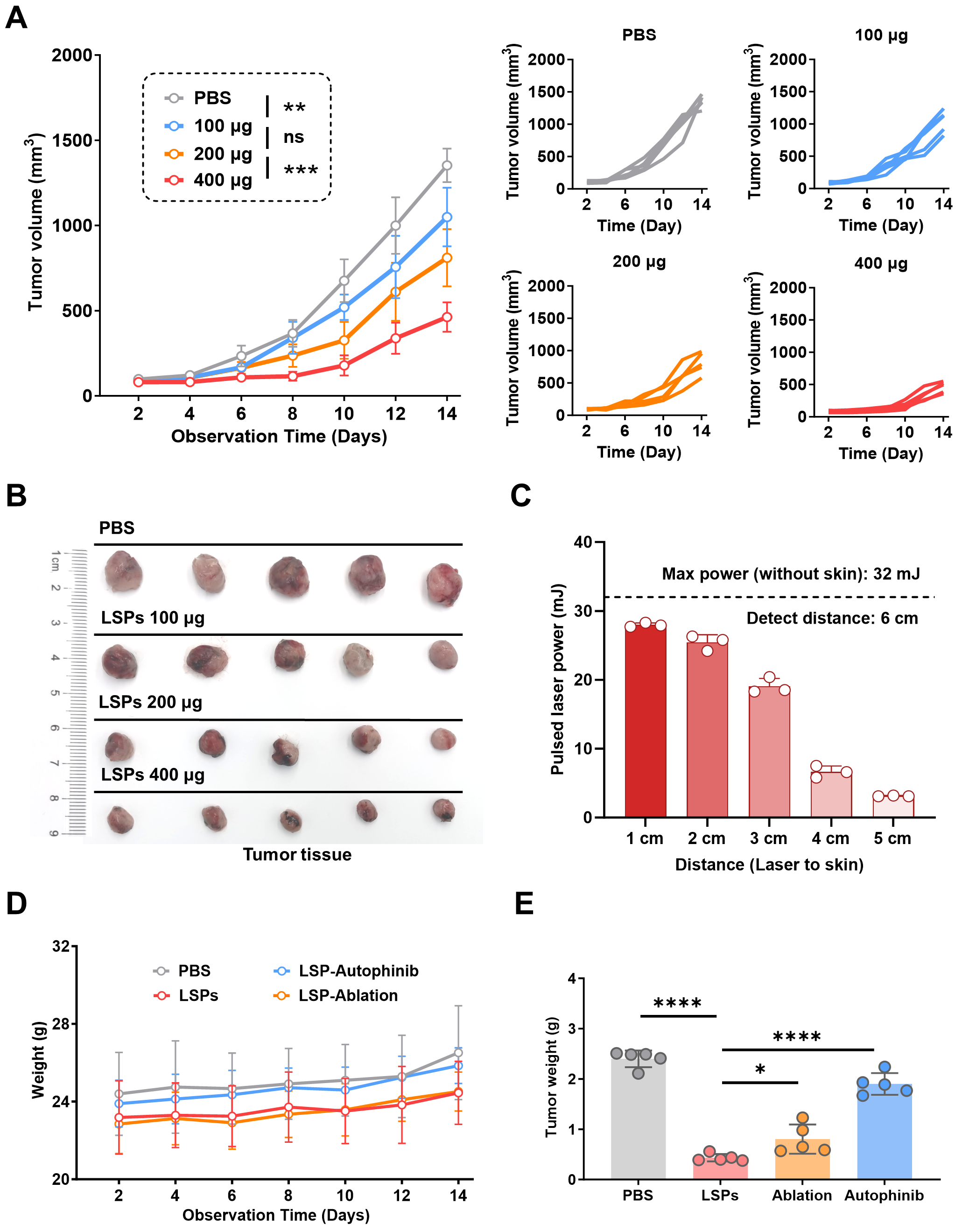


**Figure S8. *In vivo* anticancer efficacy of LSPs and ablated LSPs.** A, B) LLC cells were subcutaneously implanted into C57BL/6 mice. Mice were treated with LSPs containing iron of 100, 200, or 400 μg, and tumor growth was monitored over time (A). 14 days after LSPs treatment, tumors were dissected (B). Data are presented as mean ± s.d. Statistical significance: p < 0.05 (*), p < 0.01 (**), p < 0.001 (***), p < 0.0001 (****). C) Attenuation of pulsed laser power after penetration through mouse skin at different distances. D, E) LLC cells-bearing mice were treated with LSPs alone, LSPs combined with Autophinib, or ablated LSPs. Tumor growth kinetics (D) and final tumor weights (E) were recorded. Data are presented as mean ± s.d. (n = 5 per group). Data are presented as mean ± s.d. Statistical significance: p < 0.05 (*), p < 0.01 (**), p < 0.001 (***), p < 0.0001 (****).


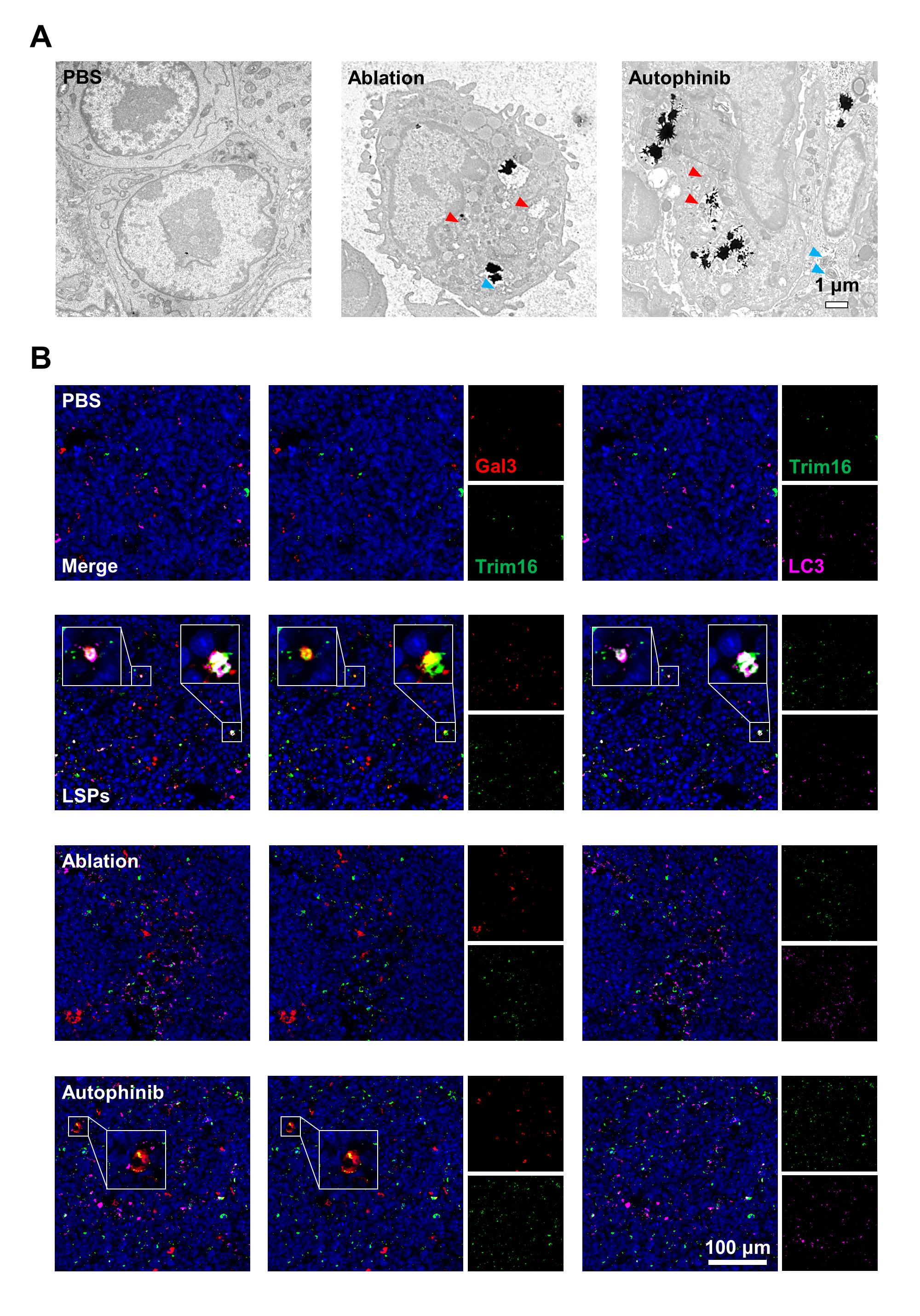


**Figure S9. Changes in autophagy levels of LLC tissues after different treatments.** A) Bio-TEM images of LLC tumor tissues treated with LSPs, LSPs plus laser ablation, or Autophinib. Red arrows indicate autolysosomes, and blue arrows indicate autophagosomes. B) Immunofluorescence staining of Gal3 (red), Trim16 (green), and LC3 (purple) in tumor tissue sections treated with LSPs, laser ablation, or Autophinib.

**
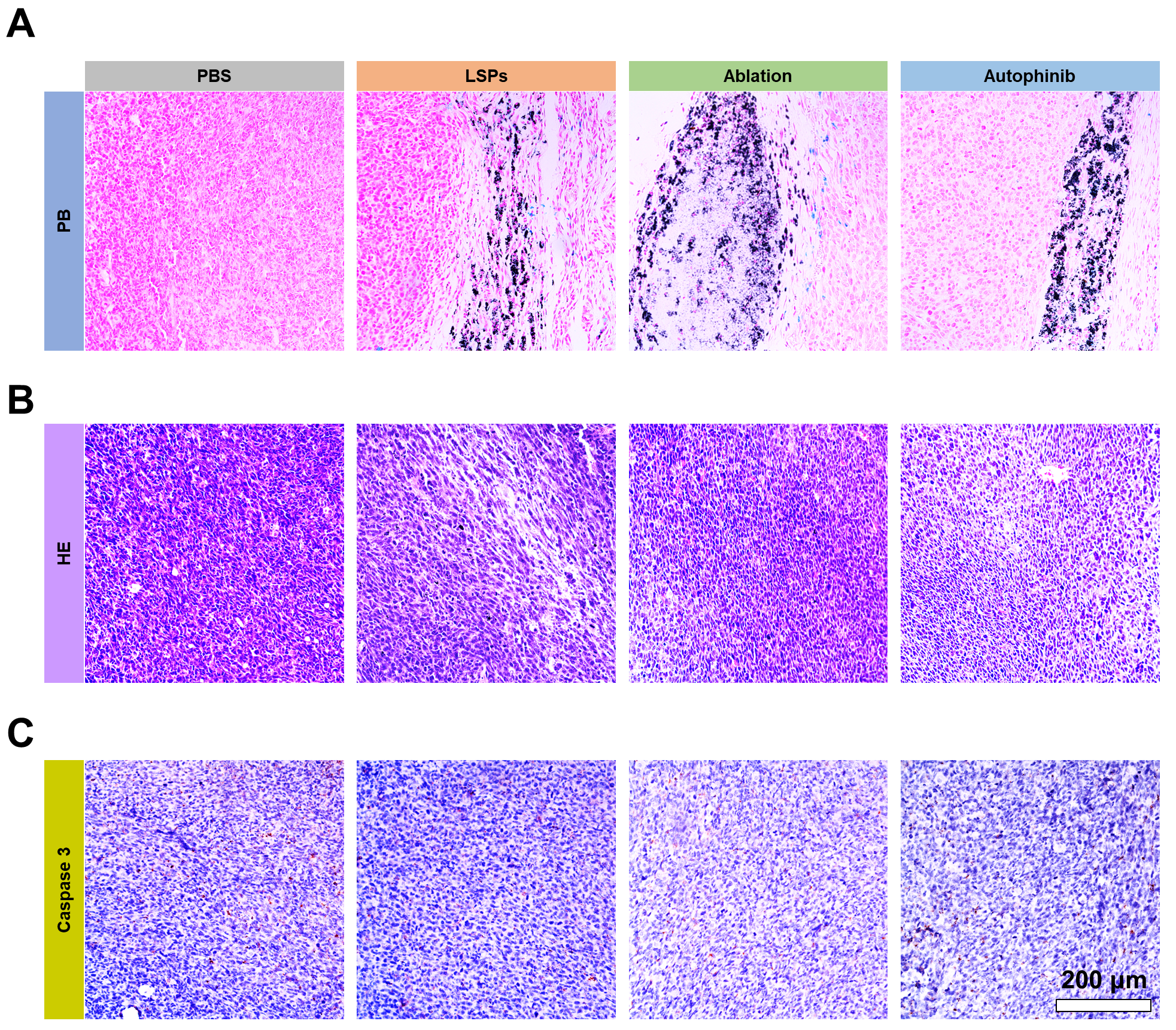
**

**Figure S10. Histological evaluation of LLC tissues after different treatments.** A-C) Representative images of Prussian Blue (A), H&E (B), and Caspase-3 staining (C) of tumor sections from mice treated with PBS, LSPs, LSPs plus laser ablation, or LSPs with Autophinib for 14 days.

**
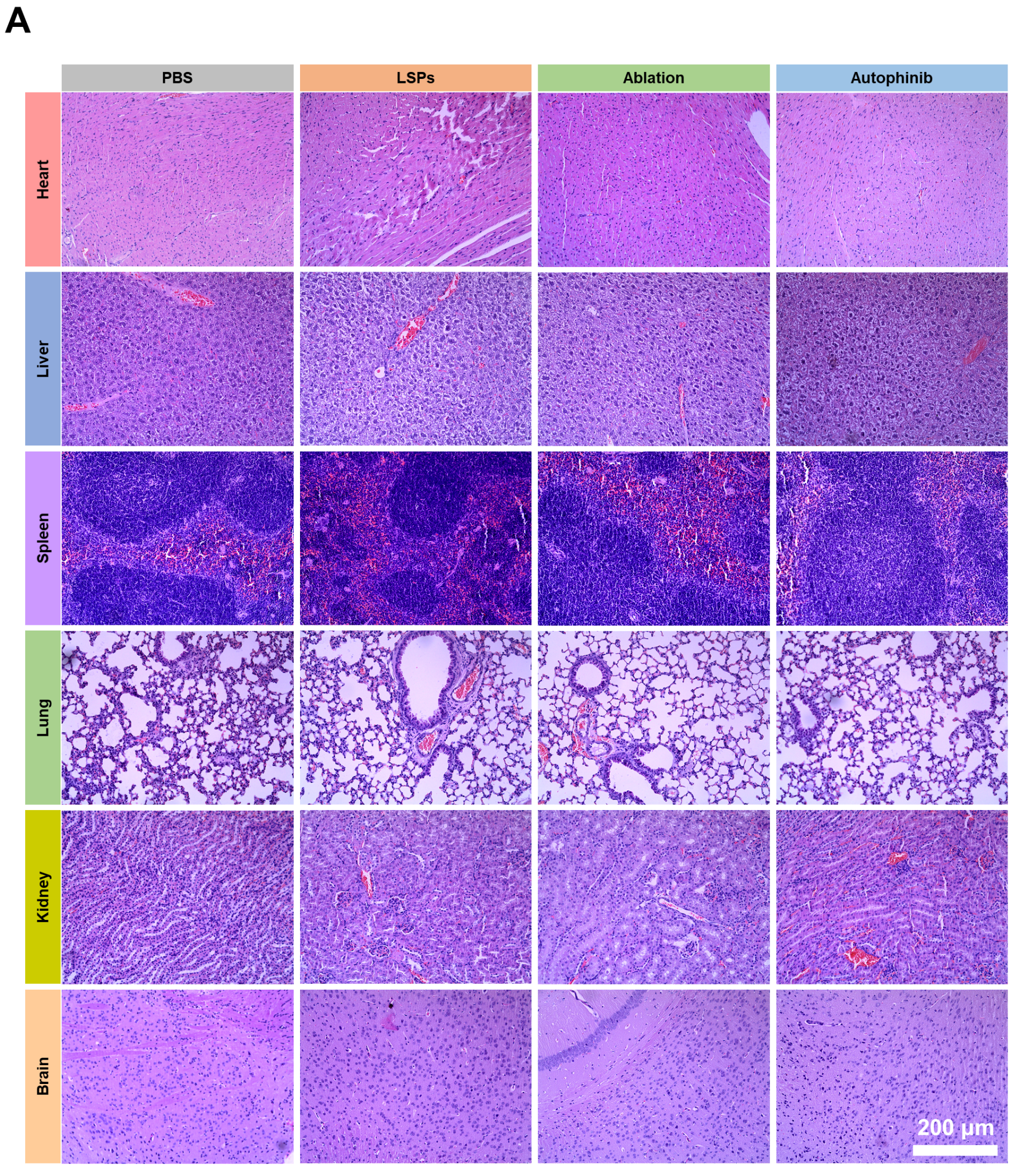
**

**Figure S11. Histological evaluation of major organs in treated mice.** A) H&E staining of the heart, liver, spleen, lung, kidney, and brain from mice treated with PBS, LSPs, LSPs with ablation, or LSPs with Autophinib for 14 days.
